# The Transcription Factor *Ultraspiracle* Influences Honey Bee Social Behavior and Behavior-Related Gene Expression

**DOI:** 10.1371/journal.pgen.1002596

**Published:** 2012-03-29

**Authors:** Seth A. Ament, Ying Wang, Chieh-Chun Chen, Charles A. Blatti, Feng Hong, Zhengzheng S. Liang, Nicolas Negre, Kevin P. White, Sandra L. Rodriguez-Zas, Craig A. Mizzen, Saurabh Sinha, Sheng Zhong, Gene E. Robinson

**Affiliations:** 1 Neuroscience Program, University of Illinois at Urbana-Champaign, Urbana, Illinois, United States of America; 2 Department of Cellular and Developmental Biology, University of Illinois at Urbana-Champaign, Urbana, Illinois, United States of America; 3 Department of Bioengineering, University of Illinois at Urbana-Champaign, Urbana, Illinois, United States of America; 4 Department of Computer Science, University of Illinois at Urbana-Champaign, Urbana, Illinois, United States of America; 5 Department of Statistics, University of Illinois at Urbana-Champaign, Urbana, Illinois, United States of America; 6 Institute for Genomics and Systems Biology, Department of Human Genetics, The University of Chicago, Chicago, Illinois, United States of America; 7 Department of Animal Sciences, University of Illinois at Urbana-Champaign, Urbana, Illinois, United States of America; 8 Institute for Genomic Biology, University of Illinois at Urbana-Champaign, Urbana, Illinois, United States of America; 9 Department of Entomology, University of Illinois at Urbana-Champaign, Urbana, Illinois, United States of America; Arizona State University, United States of America

## Abstract

Behavior is among the most dynamic animal phenotypes, modulated by a variety of internal and external stimuli. Behavioral differences are associated with large-scale changes in gene expression, but little is known about how these changes are regulated. Here we show how a transcription factor (TF), *ultraspiracle* (*usp*; the insect homolog of the Retinoid X Receptor), working in complex transcriptional networks, can regulate behavioral plasticity and associated changes in gene expression. We first show that RNAi knockdown of USP in honey bee abdominal fat bodies delayed the transition from working in the hive (primarily “nursing” brood) to foraging outside. We then demonstrate through transcriptomics experiments that USP induced many maturation-related transcriptional changes in the fat bodies by mediating transcriptional responses to juvenile hormone. These maturation-related transcriptional responses to USP occurred without changes in USP's genomic binding sites, as revealed by ChIP–chip. Instead, behaviorally related gene expression is likely determined by combinatorial interactions between USP and other TFs whose *cis*-regulatory motifs were enriched at USP's binding sites. Many modules of JH– and maturation-related genes were co-regulated in both the fat body and brain, predicting that *usp* and cofactors influence shared transcriptional networks in both of these maturation-related tissues. Our findings demonstrate how “single gene effects” on behavioral plasticity can involve complex transcriptional networks, in both brain and peripheral tissues.

## Author Summary

Animals use behavior as one of the principal means of meeting their basic needs and responding flexibly to changes in their environment. An emerging insight is that changes in behavior are associated with massive changes in gene expression in the brain, but we know relatively little about how these changes are regulated. One important class of gene regulators are transcription factors (TF), proteins that orchestrate the expression of tens to thousands of genes. We discovered that ultraspiracle (USP), a TF previously known primarily for its role in development, regulates behavioral change in the honey bee; and we show that USP causes behaviorally related changes in gene expression by mediating responses to an endocrine regulator, juvenile hormone. We present evidence that these effects on gene expression occur through combinatorial interactions between USP and other TFs, and that these hormonally related transcriptional networks are preserved between two tissues with causal roles in behavioral plasticity: the brain and the fat body, a peripheral nutrient-sensing organ. These results suggest that behavior is subserved by complex interactions between genes and gene networks, occurring both in the brain and in peripheral tissues. More generally our results suggest that molecular systems biology is a promising paradigm by which to understand the mechanistic basis for behavior.

## Introduction

Many studies have demonstrated that certain individual genes can exert strong influences on behavior, including naturally-occurring behavioral differences [1]–[4]. These results seemingly contrast with quantitative genetic and genomic studies, which have shown that behavioral variation usually involves multiple causal loci [5]–[7] and changes in the expression of hundreds to thousands of genes [8], [9]. Combining these perspectives leads to the idea that single genes influence behavior through their interactions with many other genes, but mechanisms linking behavior to single genes and gene networks are not well understood.

Transcriptional regulatory frameworks have already provided great insights into developmental and disease phenotypes, showing how transcription factors (TFs) originally identified through their individual effects on phenotypes work together to produce body parts [10] and how their dysregulation can lead to cancer [11]. Recently, large-scale genetic and genomic studies have begun to model the genome-scale transcriptional regulatory networks underlying behavior [5], [6], [12], but few studies have elucidated the molecular mechanisms linking specific genes to regulatory networks underlying specific behaviors. This is largely the case even for TFs that have been clearly demonstrated to regulate behavioral change (cf. [3], [13]).

The honey bee (*Apis mellifera*) lives in complex societies characterized by multiple forms of division of labor [14]. In a honey bee colony, the queen reproduces while workers (non-reproductive females) perform all tasks related to colony growth and development. To do this efficiently, workers exhibit a division of labor; young bees work in the nest at tasks such as broodcare (“nursing”) for the first 2–3 weeks of adulthood and then shift to foraging outside the hive for nectar and pollen for the remainder of their 5–7 week life. The age at which this transition occurs, the “age at onset of foraging,” is socially regulated and depends on colony needs, signaled by pheromones produced by the queen, brood, and older workers [15], as well as other environmental and genetic factors [16]. The age at onset of foraging is a key behavioral trait in honey bees and other social insects, and has been shown to be related to a variety of other behavioral traits such as aspects of foraging performance and colony defense, and physiological traits such as lifespan and cognitive development [16].

Hormones and signaling pathways act downstream of both heritable and environmental influences on behavioral maturation [16]. Nurses and foragers differ in the expression of thousands of genes, both in the brain [9] and in the fat bodies [17], a peripheral nutrient-sensing tissue analogous to vertebrate liver and adipose tissues [18]. Environmental and hormonal factors induce some of the same changes in gene expression that occur naturally during maturation, suggesting that their effects on behavior are rooted at the transcriptional level [19]. Moreover, reconstruction of a brain transcriptional regulatory network (TRN) for behavior demonstrated that a surprisingly large fraction of maturationally-related brain gene expression in the honey bee can be accurately predicted from the expression of TFs alone [20]. These results suggest direct links between specific TFs and behavior, but roles for specific TFs have not been experimentally demonstrated.

We selected *ultraspiracle* (*usp*; the insect ortholog of the Retinoid X Receptor, *RXR* [21]) to aim for such an experimental demonstration for the following reasons. First, the USP *cis*-regulatory motif is enriched in the promoters of genes differentially expressed between nurses and foragers [22]. Second, in other insect species USP is linked to juvenile hormone (JH), an endocrine regulator of honey bee behavioral maturation and maturation-related gene expression [23]–[25]. Third, in the bee *usp* is rapidly up-regulated in the fat bodies following JH treatments, its *cis*-regulatory motif is found in the promoters of some JH-responsive genes, and the expression of some JH-related genes is influenced by *usp* RNAi [26]–[28]. Moreover, USP's vertebrate homologs (RXRs) are master regulators of metabolism and nutritional physiology, mediating responses to a variety of nutritionally-related hormones (e.g., thyroid hormone) and lipid-like molecules [29], [30]. Changes in nutritional physiology are also linked to honey bee behavioral maturation [17], [31]. However, despite its connections to hormonal and nutritional processes that influence behavioral maturation, few studies have shown a direct effect of RXR and USP on behavior in any species (c.f., [32]), and transcriptional mechanisms are unknown.

We studied *usp* transcriptional effects in the fat bodies. Although brain circuits are the most proximal location for behavioral regulation, roles for the peripheral tissues and ganglia are also well established, especially in invertebrates [33]. In bees, the effects of nutrition and JH on behavioral maturation are thought to occur in part via their effects in the fat bodies [31]. Behavioral maturation in honey bees involves coordinated changes in peripheral and neural signaling, as occurs in most animal species including during human puberty [34]. The increase in circulating levels of JH in honey bees causes changes in endocrine gland size and function as well as changes in behavioral responsiveness to task-related stimuli [16]. Poor nutrition causes bees to initiate foraging precociously [35], and the fat bodies, the tissue responsible for lipid storage in insects, are an important sensor for nutritional changes in other insect species [36], [37], and likely in bees as well. Bees lose 50% of their lipid stores as part of normal maturation [38], and experimental inhibition of lipid storage induces precocious foraging similar to food deprivation [39]. JH treatments also cause precocious foraging [40], [41] and JH titers rise naturally prior to the onset of foraging [42], [43], whereas removal of the JH-producing corpora allata glands delays foraging ontogeny [44]. JH action influences many tissues, including the brain [19], [45], [46], and in addition a role for the fat bodies is indicated by the interactions of JH with the yolk protein vitellogenin (Vg). Vg is a conserved yolk protein that is produced exclusively in the fat bodies and that has taken on novel hormone-like functions in honey bees, including a regulatory role in behavioral maturation explicitly linked to JH [4], [47]–[49]. Together, these results suggest that the fat bodies have causal, integrative functions in the regulation of behavioral maturation, mediating responses to both nutritional status and JH.

We show that RNAi fat body knockdown of *usp* delays the age at which bees initiate foraging. We then used a combination of transcriptomics, chromatin immunoprecipitation—genomic tiling microarrays (ChIP-chip), and informatics to elucidate transcriptional targets of USP in the fat bodies and transcriptional regulatory network reconstruction in both fat body and brain to gain further insights into how USP and its targets might interact. Our results support the hypothesis that the basis for the *usp* behavioral and transcriptomic effects involve interactions with transcriptional cofactors to mediate responses to JH in both the fat bodies and brain.

## Results

### *ultraspiracle* influences behavioral maturation

**Figure 1:**
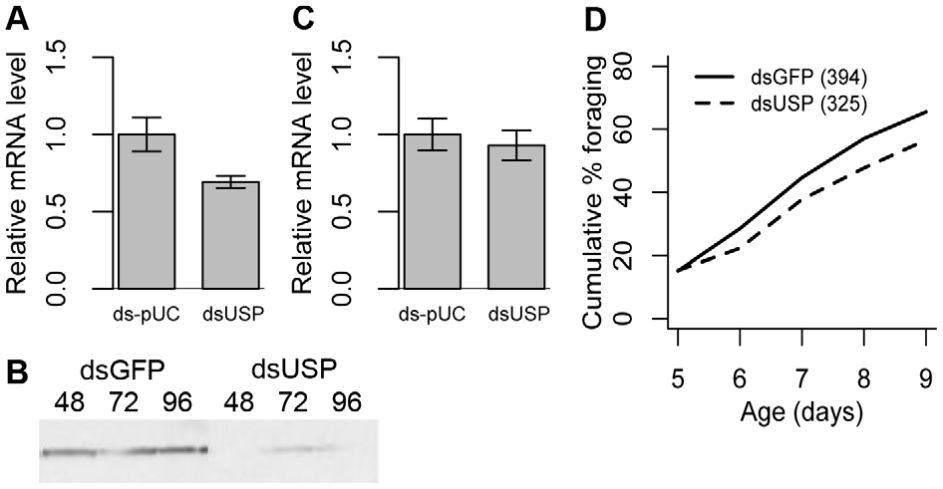
The transcription factor *ultraspiracle (usp)* influences honey bee behavioral maturation. A) Knockdown of *usp* mRNA in the fat bodies of 3-day-old bees, 72 h after injection with dsUSP or control dsRNA (ds-pUC). qPCR; n = 10 bees; t-test: * P<0.05. B) *usp* RNAi does not knock down *usp* mRNA in the heads of these same bees. C) Knockdown of USP protein in the fat bodies 48, 72 and 96 h after injection was confirmed by immunoblotting with an antibody specific to honey bee USP (Figure S3). D) *usp* RNAi delays the age at onset of foraging. Pooled results from 9 independent trials. Cox Proportional Hazards: P = 0.03. Numbers in legend indicate how many bees were measured for each group.

Because JH accelerates behavioral maturation, and because JH treatment up-regulates *usp* [26], we hypothesized that inhibition of *usp* would delay the onset of foraging. We focused on a potential role for *usp* in the fat bodies because of their known regulatory functions in this behavior, the JH and nutritional connections described above, and the known efficacy of fat body RNAi injections in honey bees [4]. Direct injection of dsUSP into the abdomen resulted in knockdown of *usp* transcripts and protein (*usp* RNAi) in fat body tissue (Figure 1A, 1B) but not in the brain (Figure 1C). We placed bees treated with *usp* RNAi (or control dsRNA) into experimental colonies in the field and observed the age at which they initiated foraging.

As predicted, *usp* RNAi caused a significant, ca. 15%, decrease in the number of bees initiating foraging during the 5 d observation period, across 9 independent trials (Cox Proportional Hazards, P = 0.03; Figure 1D). Similarly, a previous study showed that removal of the JH-producing corpora allata glands delayed but did not block foraging ontogeny [44], suggesting that molecular components of JH signaling affect the timing of behavioral maturation, and not its overall occurrence.

We also found replicable differences in the strength of the *usp* RNAi effect for bees from different genetic sources (Figure S1), suggesting that there is naturally occurring genetic variation for sensitivity to *usp*; similar genetic variation has been reported for the effects of JH analog treatments [50]. Several considerations suggest that our results were not due to a toxic effect of RNAi. First, the behavioral effect was in the opposite direction to effects of stressors: e.g., parasite infection [51], social isolation [52], or injection alone [4], each of which leads to precocious foraging. Second, 50% of the treated bees did forage during the observation period, just later than the untreated bees. Our results demonstrate that *usp* has a causal effect on honey bee behavioral maturation.

### *ultraspiracle* influences the expression of behaviorally related genes

We hypothesized that *usp* influences behavior by regulating a network of direct and indirect target genes in the fat bodies. We first characterized *usp*-responsive genes through direct mRNA sequencing of fat tissue from bees treated with *usp* RNAi. Because USP's vertebrate homologs are known to have hormone-dependent effects on transcription (reviewed in [53]), we measured the effects of *usp* RNAi in both a low hormone condition – i.e., in the presence of endogenous JH only – and in a high hormone condition, following treatment with the JH analog (JHA) methoprene (a 2×2 factorial experiment).

**Figure 2:**
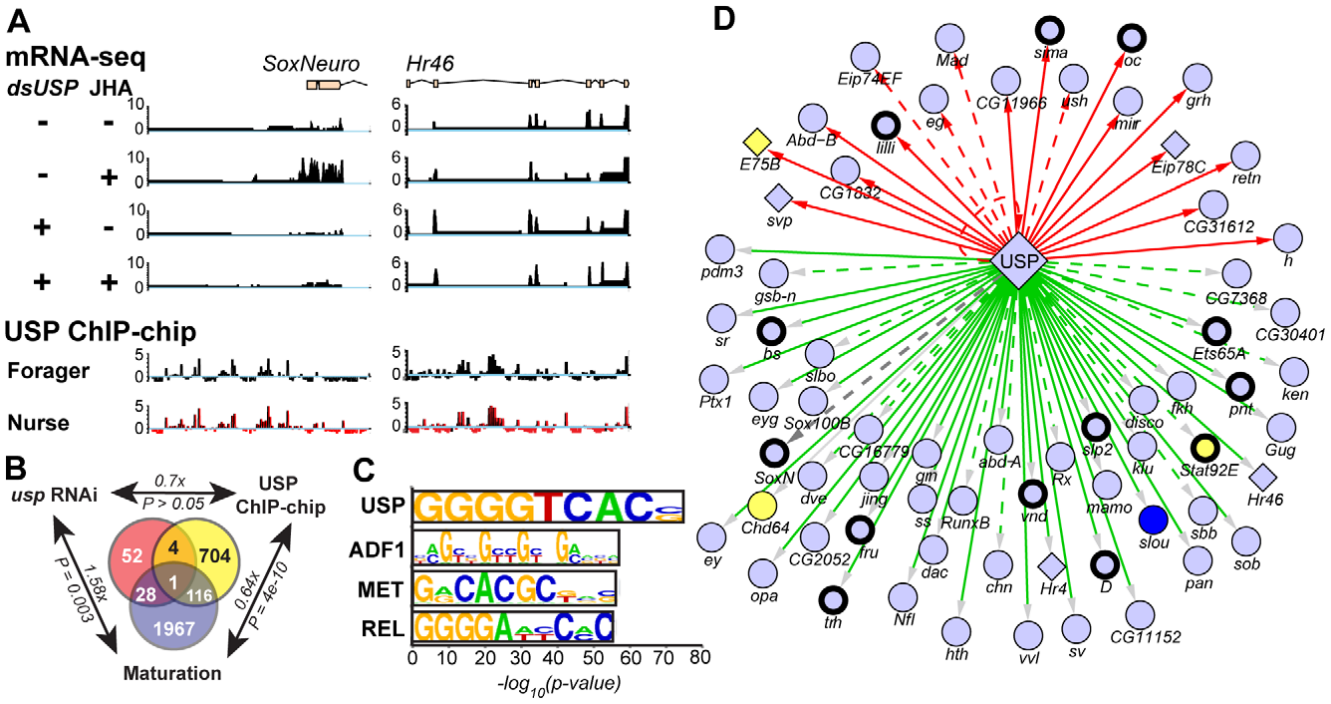
Putative direct and indirect targets of USP in honey bee fat bodies. Putative targets of USP in the fat bodies were characterized both by *usp* RNAi—deep mRNA sequencing (in combination with juvenile hormone analog, JHA, treatments) as well as by USP ChIP-chip with fat body tissue samples from nurses and from foragers. A. Genomic regions surrounding two putative target genes, the transcription factors *SoxNeuro* and *Hr46*. Units for mRNA-seq are read counts, and for ChIP-chip the ratio of a-USP to control. B. Venn diagram shows that many USP target genes identified by *usp* RNAi and USP ChIP-chip are differentially expressed between nurses and foragers (“Maturation”). Fold enrichment of overlap and its significance (hypergeometric test) are indicated for each comparison. C. The 1360 genomic binding sites of USP are enriched for conserved *cis*-regulatory sequences, putatively recognized by the TFs shown at left. D. USP binds genomic locations near 67 transcription factors (TFs), including members of the nuclear hormone receptor family (diamonds) and other TF families (circles). Some of these TFs were differentially expressed between nurses and foragers (blue, higher in nurse; yellow, higher in forager), and predicted targets for several of these TFs based on transcriptional regulatory network analysis [20] were enriched for genes that are differentially expressed in maturation-related contexts (thick outlines). Some of these TFs were also identified as USP targets in *D. melanogaster* [55] (red lines), and some binding sites contain the GGGGTCACS cis-regulatory sequence recognized by USP in *D. melanogaster* (solid lines).

Transcriptome profiling revealed 85 *usp*-responsive genes in the fat bodies (False Discovery Rate [FDR]<0.1; Figure 2A; Table S1). We confirmed 4 of these results by qPCR (Figure S2). All but 3 of the 85 *usp* RNAi-responsive genes responded statistically indistinguishably (FDR>0.1) to *usp* RNAi in the two hormone conditions, suggesting that endogenous levels of JH (or other factors) were sufficient to induce transcriptional responses of these *usp* targets.

Integrating these findings with other transcriptomics experiments [17] we found that 34% of *usp* targets were among those that we previously found to be differentially expressed in the fat bodies of nurses and foragers (29 of 85; 1.58x-enriched, P = 0.003; Figure 2B). These results suggest that *usp* regulates behavior at least in part by influencing (directly or indirectly) the transcription of maturationally-related genes in the fat bodies.

We suspect that this list of 85 *usp* RNAi-responsive genes represents only a fraction of *usp*'s targets, for the following reasons. RNAi resulted in a modest knockdown of *usp* (ca. 35%), and we measured only a single time point (72 h after dsRNA injection). We also likely missed genes that respond to *usp* only under specific hormonal or nutritional conditions not present in this experiment; context-dependent responses to USP are well-known in other species [53].

### USP binds genomic regions near behaviorally related genes

Seeking to identify a broader set of putative direct target genes, we characterized USP's genomic binding sites with ChIP-chip, in 6 independent replicates using tissue from the fat bodies of nurses and foragers (ChIP was performed using an antibody specific to honey bee USP; Figure S3).

This experiment revealed 1360 genomic binding sites for USP (Figure 2A; Table S2; ChIP-qPCR validation for a few binding sites shown in Figure S4). These sites were located within 10 kb of 848 putative direct target genes (Table S2). 759 binding sites (53%) contained at least one copy of a well-characterized *cis*-regulatory DNA sequence bound by USP in *Drosophila melanogaster*; this was the most strongly enriched motif out of >600 motifs we examined (the “GGGGTCACS” motif [54]; P<1e-75; Figure 2C). In addition, 182 of these 848 putative target genes are also located within 10 kb of a USP binding site in the *Drosophila* genome [55]. (By these criteria, 5643 genes were considered putative targets of USP in *Drosophila*, of which 3068 had bee orthologs. The 182 target genes shared between the two species represents 45% of the bee targets with one-to-one orthologs but was not statistically enriched; P>0.05). The combination of conserved *cis*-regulatory sequences and conserved target genes suggests extensive evolutionary conservation of the USP regulatory network, despite ca. 300 M years divergence between bees and flies.

It appears that many putative direct USP target genes are involved in maturation. USP targets included components of signaling pathways that have previously been shown to influence the timing of behavioral maturation, such as *inR1* (a receptor for insulin-like peptides [56]) and *foraging* (a cGMP-dependent protein kinase [57]). 117 of the 848 putative direct USP target genes (14%) were differentially expressed between nurses and foragers (Figure 2B; this subset was not statistically enriched: P>0.05). These results suggest that *usp* influences maturation through a subset of its direct targets, including several genes already known to function in behavioral maturation, but which had not been known to work together. That we did not find stronger overlap between USP targets and genes differentially expressed between nurses and foragers is not necessarily surprising; USP is known in other species to regulate distinct sets of targets via interactions with different cofactors [53], so different subsets of USP targets are likely active in other contexts.

Our experiments have identified both putative direct and indirect targets of USP that are components of a hierarchical transcriptional network underlying maturation. Five of the 85 genes that responded to *usp* RNAi were located within 10 kb of one of USP's genomic binding sites (Figure 2B; Table S1); these 5 are likely direct targets. The remaining 80 genes are more likely indirect targets.

Further evidence for a hierarchical structure of the USP regulatory network comes from the enrichment of USP's direct targets for other TFs (Gene Ontology, “regulation of transcription”, *P*<3.02e-9), including the bee homologs of 67 putative TFs from the FlyTF database (Figure 2D). Some of these TFs are likely the direct regulators of USP's indirect targets, and they include TFs known to function in hormonal signaling cascades (e.g., *Hr46* [Figure 2A], *E75*, *Chd64*, and *usp* itself [25], [46], [58]) and in the regulation of behavior (e.g., *fruitless* and the *Egr* homolog *stripe*; reviewed in [59]). Two of these TFs among USP's targets – *E75* (a nuclear hormone receptor critical for responses to JH during development [25]) and *Chd64* (which physically interacts with USP as part of a protein complex bound to JH response elements [24]) – were up-regulated in foragers compared to nurses (Figure S5), so these JH-related TFs may be particularly likely to function in feed-forward regulation of maturationally-related targets of USP. A third TF, *SoxNeuro* (Figure 2A), was one of the few genes identified as a USP target by both ChIP-chip and transcriptomics, suggesting that it too could have an integral role in downstream responses to USP.

Together, *usp* RNAi, transcriptomics, and ChIP-chip revealed direct and putatively indirect USP targets in the honey bee fat bodies, including a large fraction that were differentially regulated during maturation. We next explored hormonal, nutritional, and transcriptional mechanisms linking USP target genes to behavior.

### *usp* mediates behaviorally related transcriptional responses to juvenile hormone

**Figure 3:**
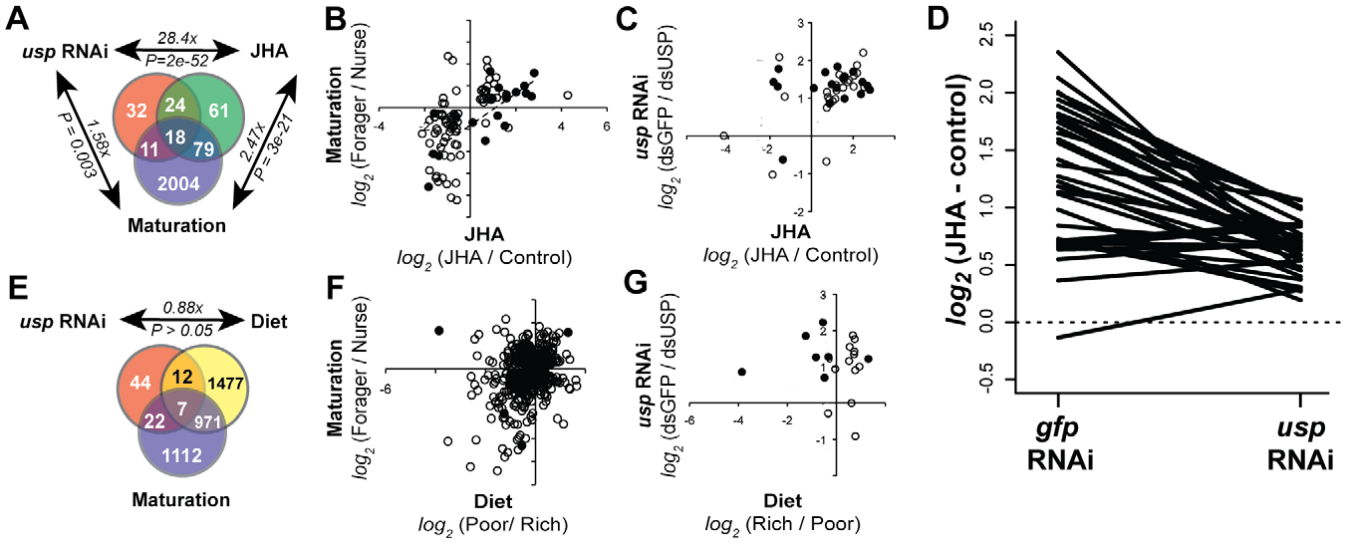
USP mediates maturation-related gene expression responses to juvenile hormone. Mechanisms linking USP's putative targets to maturation were examined by studying their expression dynamics during maturation and in response to juvenile hormone analog treatments (JHA) and diet manipulations. A. *usp* RNAi, JHA, and maturation influence many of the same genes. Fold enrichment of overlap between gene lists and its hypergeometric p-value are indicated for each comparison. B. Fold change responses to JHA and maturation are positively correlated. Data are shown for 97 genes that responded significantly to both JHA and maturation; genes that also responded to *usp* RNAi are represented by closed circles. C. 33 of the 42 genes that respond to both JHA and *usp* RNAi were activated by both factors. Genes that also responded to maturation are represented by closed circles. D. *usp* RNAi inhibited transcriptional responses to JHA. Fold responses to JHA in control (*gfp* RNAi) and *usp* RNAi conditions are shown for each of the 33 genes that were activated by both USP and JHA. E. Few *usp* RNAi-responsive genes are regulated by diet quality. F. Fold change responses to diet quality and maturation are weakly correlated (978 genes that responded significantly to both). G. Fold change responses to *usp* RNAi and diet quality are uncorrelated (19 genes that responded significantly to both; genes that also responded to maturation are represented by closed circles).

We explored the hypothesis that *usp* mediates responses to JH and nutritional status by integrating our results with additional transcriptomics experiments. We identified 182 genes in the fat bodies that were differentially expressed in response to JH analog treatment (based on the *usp* RNAi x JHA mRNA-seq factorial experiment described above; Figure 3A, Figures S6 and S7; Table S3). Most JHA-responsive genes were also differentially expressed between nurses and foragers (97 of 182 genes; Figure 3A), and their responses to JHA and maturation were strongly correlated (r = 0.57; P = 9e-10; Figure 3B). JH thus induces “forager-like” gene expression in the fat bodies, as in the brain [19].

Our results indicate a close association between transcriptional responses in the fat bodies to USP, JH, and maturation. Nearly half of the 85 *usp*-responsive genes (42 genes; 47%) also responded to JHA (P = 6.1e-54), including 18 that were additionally differentially expressed between nurses and foragers (Figure 3A). Moreover, 33 of the 42 genes that responded to both *usp* RNAi and JHA (79%) were downregulated by *usp* RNAi and upregulated by JHA. Therefore, USP and JH frequently activate the same genes, including those that are involved in maturation (Figure 3C).

The overlap between transcriptional targets of USP and JHA suggested the hypothesis that they work together to regulate transcription. If USP acts downstream of JH, transcriptional responses to JHA should be inhibited by *usp* RNAi. However, as noted above, we found significant *usp* RNAi x JHA interactions (FDR<0.1) for only 3 of 85 *usp* RNAi-responsive genes. Our inability to detect statistical interactions could relate to technical limitations such as incomplete *usp* RNAi knockdown. Alternatively, USP and JH may independently regulate their shared transcriptional targets.

To further explore the relationship between USP and JH, we focused on the 33 genes that were activated by both. Indeed, 27 of these 33 genes had smaller fold responses to JHA following *usp* RNAi (Figure 3D). For instance, the response of *SoxNeuro* to JHA was almost completely blocked by *usp* RNAi (Figure 2A). The predominance of decreased vs. increased fold changes was statistically significant (P = 6e-4) and was not an artifact of a bias in the broader dataset (Figure S8). Taken together, these results support the hypothesis that USP mediates transcriptional responses to JH. We cannot discern if these effects of USP on JH signaling are direct or indirect.

We performed similar analyses to determine whether *usp* is also involved in the effects of nutritional status on behavioral maturation. We showed in a previous study that the fat bodies of bees fed nutrient-rich vs. nutrient-poor diets differ in the expression of 3372 genes, about half of which were also differentially expressed between nurses and foragers [17] (Figure 3D). In contrast to our findings for maturation- and JHA-responsive genes, there was no significant overlap between *usp*-responsive and diet-responsive genes (19 genes, 0.88x-depleted, P = 0.08; Figure 3D, 3E). There was also no apparent bias in the directions in which *usp* and diet influenced these genes (11 genes regulated in directions concordant with the effects of *usp* RNAi and diet quality on maturation; 8 genes discordant; Figure 3F). Therefore, *usp* likely acts downstream of JH, but not nutritional status, in regulating behavioral maturation.

### USP genomic binding does not differ between nurses and foragers

By what mode of action does USP mediate responses to JH and influence behavioral maturation? In other species, USP (i.e., RXR) most frequently regulates transcription via interactions with additional TFs with which it forms heterodimers, typically activating gene expression only when a hormone or other ligand is bound to one of these cofactors [53]. The ability of USP to interact with several different TFs could also explain why only a subset of USP targets in the bee are associated with maturation. We performed additional informatic analyses to explore potential mechanisms linking USP and transcriptional cofactors to JH and maturation.

**Figure 4:**
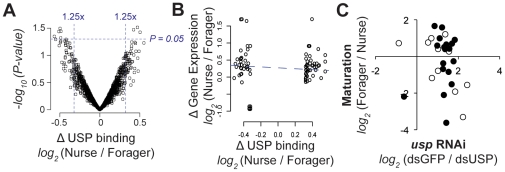
USP binds to similar genomic locations in nurse and forager fat bodies. The genomic binding sites of USP were assessed in 3 biological replicates each from nurse and forager fat bodies. A. Comparison of the intensity with which USP bound each of its 1360 binding sites in nurses and foragers. Normalized, *log2-*transformed fold differences in binding intensity and the significance of these differences (ANOVA) are shown. We observed no dramatic differences in USP binding at any of these sites between nurse and forager samples. B. Differences in USP binding between nurses and foragers were uncorrelated with the expression of nearby genes in nurses and forager fat bodies. Data are shown for 116 genes located near genomic binding sites of USP that had >1.25-fold difference in binding intensity between nurses and foragers. C. Fold change responses to *usp* RNAi and maturation are weakly correlated. Data are shown for the 29 genes that responded significantly to both. Each circle represents a single gene. Genes represented by closed circles also responded to juvenile hormone analog treatments.

In its most common mode of action, USP binds the same genomic locations regardless of hormonal titers [53]. Several results suggest that differential binding of USP in the genomes of nurses and foragers is not an important mechanism by which this TF influences behavioral maturation. First, there were no statistically significant nurse-forager differences in USP genomic binding sites in fat body (P>0.01, FDR>0.9 for all binding sites; no fold difference >1.46; Figure 4A). Second, we found strong correlations in binding intensity between nurse and forager samples across all 1360 binding sites (r = 0.96, P≪2.2e-16; Figure S9). Third, when we focused on the subset of USP target genes that showed even a hint of differential binding (116 genes with 1.25–1.46 fold, non-significant differences between nurses and foragers), we found no relationship between this differential binding and differential gene expression (Pearson correlation, *r* = −0.1, *P* = 0.34; Figure 4B). Fourth, although genes that responded to *usp* RNAi were often differentially expressed between nurses and foragers (above), the direction of their responses to *usp* RNAi and to maturation were only weakly correlated (Pearson correlation, r = 0.20, P = 0.29; Figure 4C). These results suggest that while USP targets are frequently involved in maturation, maturation-related changes in USP binding or expression do not determine the direction of these transcriptional responses. Since *usp* is known in other contexts to activate transcription via TF cofactors [53], [60], we next focused on characterizing potential interactions with additional TFs.

### *cis*-regulatory sequences predict JH–related transcriptional cofactors of USP

We considered two possible roles for transcriptional cofactors in USP-mediated, maturation-related, transcriptional responses to JH. First, because USP itself does not bind JH at physiological levels [23], [25], additional TFs might be involved in linking transcriptional responses to JH and USP. Second, because distinct subsets of USP targets were either high in foragers or high in nurses other TFs might be involved in distinguishing these sets of targets. We searched for molecular signatures of potential cofactors by scanning the genomic regions around USP's binding sites for matches to ca. 600 *cis*-regulatory motifs that had been identified previously in vertebrates or in *D. melanogaster* (Methods). TFs in the same family often recognize very similar motifs, so this information alone cannot assign specific cofactors for USP. Rather, this analysis was designed to test general hypotheses about classes of potential cofactors.

**Figure 5:**
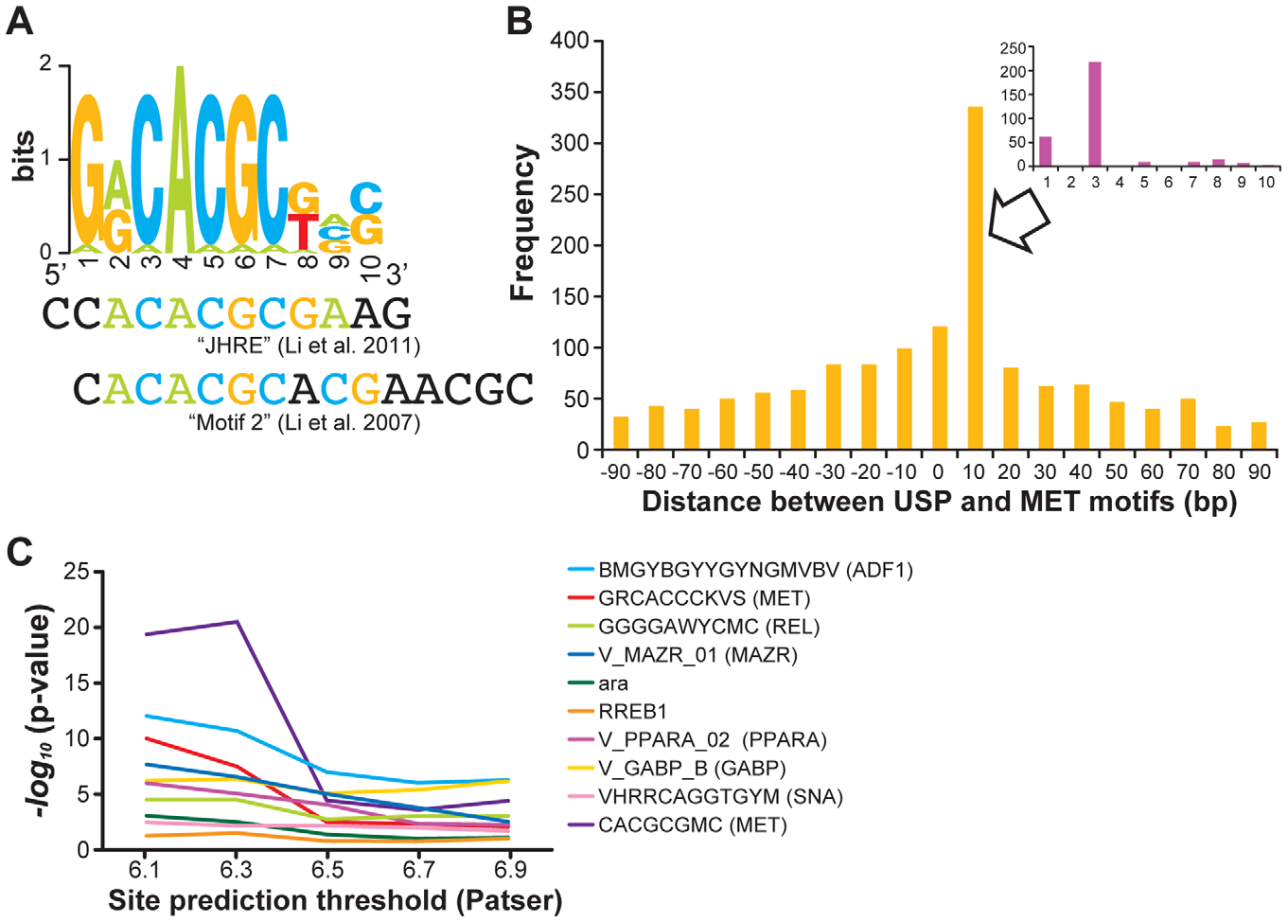
*cis*-regulatory sequences predict behaviorally-related responses of USP targets. A. The GRCACGCKVS motif enriched at USP binding sites (Figure 2C) matches a Juvenile Hormone Response Element (JHRE) recognized by MET (and other bHLH TFs) [24], [61]. B. Predicted binding sites of USP and MET (the GGGGTCACS and GRCACGCKVS motifs, respectively) were consistently overlapping, with start positions located 3 bp apart. All adjacent pairs of Patser-predicted matches to these motifs in USP-binding loci (ChIP peaks) were considered; shown is the histogram of spacing between start positions of each pair, with negative numbers indicating that the MET site is on the left. Inset shows a zoomed-in view of the histogram for spacings in the range of 1–10 bp. C. Spacing constraints between sites of GGGGTCACS (USP) and potential cofactor motifs. Each of the 10 most significantly overrepresented motifs in USP-binding loci was tested for a constraint on spacing (< = 25 bp vs. >25 bp) between sites for USP and that motif. The Y-axis shows the −log10 of the p-value of this test, at five different statistical thresholds (X-axis) for defining motif matches.

We first scanned the genomic regions around all USP binding sites to identify signatures of potential “general” transcriptional cofactors. As noted above, the strongest enrichment observed was for a USP motif (“GGGGTCACS”, P = e-75), but a handful of other motifs also appeared significantly enriched (Figure 2C). One of the most enriched motifs was “GRCACGCKVS” (P = e-56), which matches a putative Juvenile Hormone Response Element (JHRE) recognized by the bHLH TF Methoprene-tolerant (*Met*) [24], [61] (Figure 2C; Figure 5A). This motif is likely recognized by multiple members of the bHLH family of TFs, but the possibility of MET binding is particularly intriguing. This TF is required for developmental responses to JH in some insect species [62], binds JH with strong affinity [63], and has been shown to form protein-protein interactions with USP *in vitro* [64].

We also observed that putative USP binding sites (i.e., the locations of “GGGGTCACS” motifs within peak regions identified by ChIP-chip) and the nearest GRCACGCKVS motif (putative bHLH binding site) were consistently overlapping, 3 base pairs apart (Figure 5B). Closely spaced pairs of motifs can indicate physical interaction between the TFs that recognize those motifs [65], so this result supports the idea that USP and a bHLH TF such as MET work together to mediate responses to JH [25], [64]. Motivated by this observation, we designed a statistical test for spacing constraints between adjacent pairs of binding sites for USP and each of the next 10 most highly enriched motifs within peak regions (Figure 4D). Strongest evidence of such constraints was found for two motifs known to bind MET in *Drosophila*, CACGCGMC and GRCACGCKVS, and a motif recognized by ADF, BMGYBGYYGYNGMVBV, and we found significant spacing constraints (P< = e-3), for 7 out of the 10 tested motifs (including MAZR, PPAR, GABP_B, and ARA). These results indicate that USP binding sites in honey bee fat body contain sequences recognized by multiple other TFs, with a particularly strong signature for a JH-associated motif that is putatively bound by bHLH TFs.

Why do different subsets of USP targets show distinct responses during behavioral maturation? In other species, USP regulates distinct targets and can shift from activation to repression of its targets depending on which other transcription factors are present [53], [60]. To explore the idea that this might also occur in the context of behavioral maturation, we compared the *cis*-regulatory sequences present at USP binding sites near target genes with different classes of transcriptional response (high in foragers, high in nurses). This analysis revealed a variety of motifs that differentiated these binding sites. For instance, “high-foraging” binding sites often contained the PPARG_RXRA motif, recognized in vertebrates by a heterodimer of USP and PPARγ (a well-known nutritionally-related cofactor of USP [29]), but this motif was almost never present at “high-nursing” binding sites (P = 0.003) (Table S4). Insect homologs of PPARs are not known, but this result suggests that a particular USP-containing heterodimer specifies foraging-related gene expression. In addition, high-nursing binding sites were more likely than high-foraging binding sites to contain motifs recognized by the TF *broad*, which acts downstream of JH in developmental contexts [58] (“br_Z4”, P = 0.0001; “I_BRCZ3_01”, P = 0.0002). These results suggest that, as in other contexts [53], [60], USP regulates behavioral maturation by interacting with context-specific transcriptional cofactors.

### Transcriptional modules associated with behavioral maturation, *usp*, and JH are preserved between fat and brain

We have thus far described transcriptional mechanisms by which JH and *usp* influence the expression of maturationally-related genes in the fat bodies. In addition to its effects on the fat bodies, JH is known to cause behavioral maturation-related changes in brain morphology [45], brain chemistry [66], and brain gene expression [19]. If our findings do indeed reflect the effects of *usp* on behavioral maturation, we would expect that similar transcriptional mechanisms underlie hormonally-mediated maturational changes in the brain.

To explore this issue, we generated microarray transcriptome profiles from both the brains and fat bodies of 60 individual bees, collected following manipulations of nutritional and hormonal factors that are known to influence behavioral maturation in part via their action in the fat bodies: rich vs. poor diet and vitellogenin RNAi, respectively. Poor diet accelerates behavioral maturation (measured as a precocious onset of foraging [39]), and this effect is also caused by vitellogenin RNAi knockdown [4]. We used a combination of Weighted Gene Co-expression Network Analysis [67] and CoherentCluster [68] to characterize modules of genes that were tightly co-expressed with each other in both tissues. We also assembled (from previous publications and statistical analysis of our *vg* RNAi and diet microarray experiments) lists of individual genes that responded in the fat bodies and brain to maturation [17], [69], JHA [19], *vg* RNAi [17], and diet [17]. We then integrated these datasets and our list of USP targets to explore the roles of each of these factors in coordinating gene expression changes in the periphery and brain.

Our results suggest extensive co-regulation of gene expression responses in fat and brain, both for individual genes and for modules of co-expressed genes. Many genes influenced by maturation, JHA, *vg*, and diet were differentially expressed in both the fat bodies and brain, and the fold change responses of these genes to each of these factors were positively correlated between the two tissues (Figure S10).

Individual genes with coordinated responses in the two tissues included components of the JH signaling pathway, including a TF that physically interacts with USP on the promoters of JH-responsive genes, *Chd64* (a USP target detected in our analyses, above), as well as a JH-degrading enzyme, JH epoxide hydrolase [70], [71] (Figure S11). These results suggest that maturation influences many of the same genes in the fat bodies and in the brain, and that these concordant responses involve JH.

**Figure 6:**
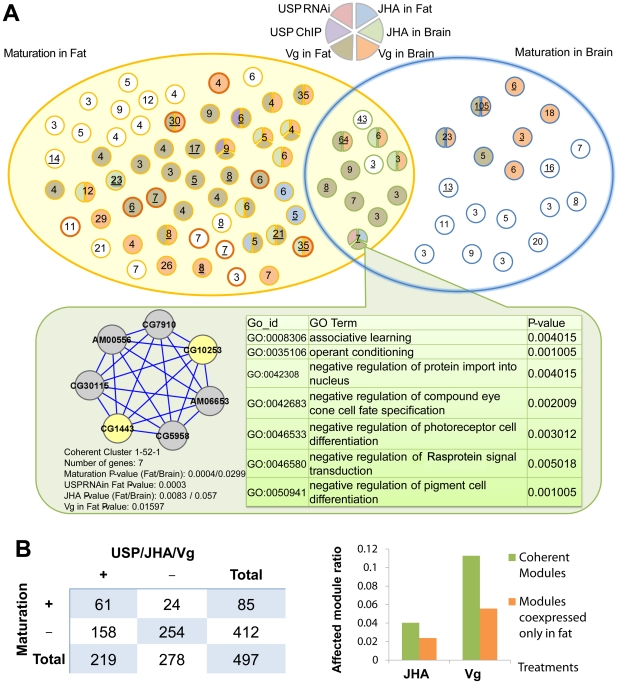
Transcriptional co-expression analysis reveals that maturation involves overlapping, hormonally regulated gene modules in the fat bodies and brain. A. CoherentCluster revealed 85 maturation-related “coherent” gene modules that were preserved between the fat bodies and brain (colored circles). These modules were enriched for genes that were differentially expressed between nurses in foragers in either the fat bodies, the brain, or both, as indicated by Venn Diagram categories. The number within each circle indicates the number of genes in the module; a bold outline indicates that the module contains at least one TF; underlined numbers indicate modules containing at least one *usp*-related gene (identified empirically by either RNAi or ChIP-chip experiments). Circles are colored based on their enrichment (P<0.05) for genes that are differentially expressed in response to *usp* RNAi, *vg* RNAi, or JHA. One module (f-15-2-1) is selected for demonstration (inset). Nodes in this inset represent individual genes, and edges indicate co-expression. Yellow nodes indicate genes that were responsive to (all three of) *usp* RNAi, *vg* RNAi, and JHA in the fat bodies. In addition, we show enriched Gene Ontology processes for genes within this module. B. Hormones regulate the expression of genes within many coherent modules. Left: contingency table for the number of coherent modules that were enriched for maturationally-regulated genes and for hormonally-regulated genes (USP targets, JHA-responsive genes, and Vg RNAi–responsive genes). Right: Proportion of coherent modules and of fat body-specific modules that were enriched for JHA-responsive genes and Vg RNAi-responsive genes.

Gene co-expression analysis suggested that these shared patterns of differential expression are due to co-regulation. We found 497 “coherent” modules of genes, i.e., modules that were tightly co-expressed with each other in both fat and brain (each module contained between 3 and 105 genes; Table S5). Much of this brain-periphery co-expression was linked to maturation: 85 of 497 coherent modules were statistically enriched for genes that were differentially expressed in one or both tissues during maturation (Figure 6A; Table S5).

Two results suggest a role for maturationally-related hormones in driving these coordinated responses. First, 61 out of 85 maturation-related modules were also enriched for genes that responded to at least one of the following – JHA, Vg RNAi, USP RNAi, or Diet – significantly more than expected by chance (P = 1.6e-8; Figure 6A, 6B). Second, modules of genes that were co-expressed with one another in only one tissue were less likely to be enriched for genes influenced by JHA and Vg than were modules of genes co-expressed in both fat and brain (Figure 6B). Together, these results suggest that shared hormonally-driven, transcriptional mechanisms underlie the coordinated peripheral and neuronal changes that occur during behavioral maturation.

## Discussion

We have shown that the transcription factor *ultraspiracle* influences both behavioral maturation and the expression of maturation-related genes in honey bees, most likely via interactions with transcriptional cofactors to mediate responses to JH, in both the fat bodies and brain. These results provide insights into the mechanisms linking social behavior to genes and gene networks.

**Figure 7:**
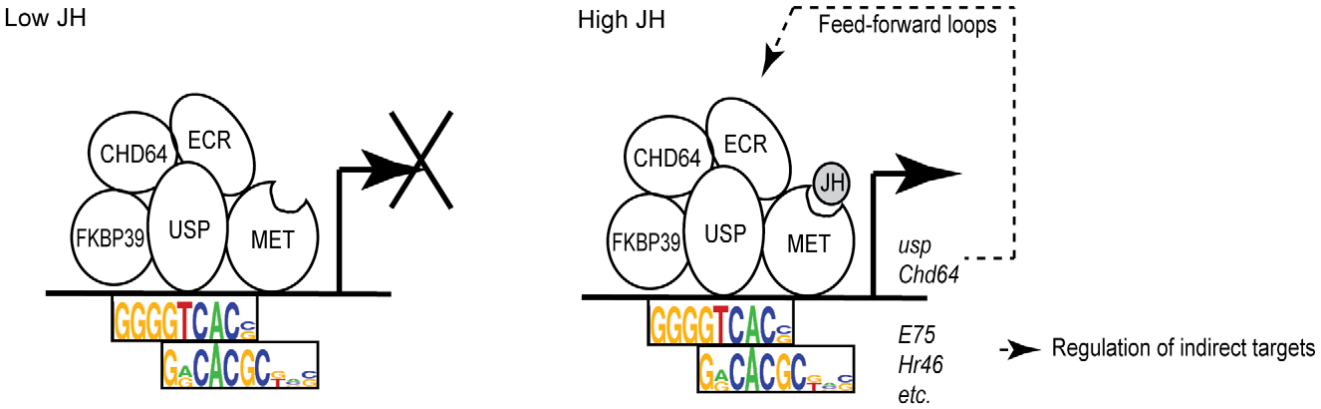
Model for *usp* regulation of behavioral maturation. According to this model, USP mediates responses to JH as part of a complex of proteins – pre-assembled at the promoters of JH-responsive genes – that likely includes USP, MET, EcR, Chd64, and FKBP39; all of these TFs have been shown to physically interact with one another *in vitro* [24], [64]. Low JH titers (nurse bees) lead to target gene repression. High JH titers (foragers) cause target gene activation. This might occur via ligand-dependent conformational changes in the protein complex and recruitment of general transcriptional machinery, both of which are known for USP in other contexts [53] (not shown). JH most likely binds MET, the only TF in this complex known to have strong affinity for JH [63]. The model also suggests the presence of a feed-forward loop [10] that stabilizes responses to JH; this role could be played by two components of the JH signaling complex – *usp* and *Chd64* – themselves USP targets. Other TFs among USP targets – including TFs previously implicated in JH signaling such as *E75* and *Hr46* [25] – are available to propagate these responses to indirect targets of USP and JH. This model can explain differential gene expression caused by both USP and JH despite the fact that USP was found to bind the same genomic locations in the fat bodies of nurses and foragers. It also is consistent with findings that these genomic locations are enriched for two very closely located (3 bp apart) *cis*-regulatory motifs, one recognized by USP and the other recognized by bHLH TFs, including MET.

Our results fall short of final proof for any specific mechanism for USP's influence on behavioral maturation, yet our evidence, together with prior results from JH experiments [24], [25], [58], [61]–[64], support the conclusion that the effects of this hormone are mediated by a complex transcription-based mechanism involving USP. We present a verbal model (Figure 7) for the largest class of USP gene targets detected in this study, which were down-regulated by *usp* RNAi and up-regulated by JHA. We propose that USP mediates responses to JH as part of a complex of proteins pre-assembled at the promoters of JH-responsive genes. This model can explain differential gene expression caused by both USP and JH despite the fact that USP was found to bind the same genomic locations in the fat bodies of nurses and foragers.

Our model builds on existing knowledge about the modes of action of both USP and JH. In other species, USP forms heterodimers with other TFs to mediate responses to a variety of hormones and other lipid-soluble molecules [53], [72]. These complexes of TFs are often pre-assembled at promoters and only influence transcription when hormones or other ligands are present [53]; this is consistent with our finding that USP binds in the same genomic locations in nurses and foragers. USP is known to regulate distinct sets of target genes by interacting with several different heterodimer partners [53], [55], which may explain why only a subset of USP targets were associated with honey bee maturation. Moreover, interactions between USP and other TFs in other species have been shown to cause some USP targets to be up-regulated and others down-regulated in response to the same hormone [60]. The distinct *cis*-regulatory motifs surrounding nursing-related and foraging-related targets of USP hint at a similar mechanism underlying transcriptional responses to JH during honey bee maturation. Together, these results suggest that USP functions in honey bee maturation by mechanisms similar to those known in other species for other USP-influenced phenotypes.

JH has long been known to regulate behavioral maturation in honey bees [37]–[41] and other forms of behavioral plasticity in insects [25], but underlying molecular mechanisms for its behavioral effects have remained elusive. Our study adds to a growing body of evidence that a complex of proteins including USP, MET, EcR, Chd64, and FKBP39 mediates responses to JH [24], [25], [64]. A central role for MET in this complex is supported by its ability to bind physiological concentrations of JH [63], and manipulations of MET influence JH-related developmental phenotypes in a variety of insect species [25], [61], [62]. Our study suggests that USP is also required for normal JH signaling, at least in the context of worker honey bee behavioral maturation. A previous study showed that USP and MET physically interact *in vitro* and *in vivo* [64]. Our discovery of co-localized *cis*-regulatory motifs recognized by USP and bHLH TFs provides further evidence that these TFs could work together to mediate responses to JH. Most known heterodimer partners of USP are other members of the Nuclear Hormone Receptor family of TFs, but MET is a bHLH TF. Thus, if our hypothesized interaction between USP and MET proves true, the JH signaling complex has similarities to the USP-containing heterodimers that mediate responses to other hormones but is an atypical variation on that theme.

Our study provides a first attempt to experimentally characterize the transcriptional regulatory mechanisms underlying division of labor in honey bee colonies, but many questions remain, both about the roles of USP and of other TFs. Our ability to draw definitive conclusions about USP is limited by the incomplete RNAi knockdown of USP that occurred in our study. Would a stronger knockdown have led to a longer delay in foraging ontogeny, or influenced the expression of more genes, or completely block responses to JH? We expect that these questions will be answered in the future as technology for genetic manipulation in honey bees improves. We also anticipate that some of the questions about the role of USP in JH signaling raised by this study can be addressed immediately in *Drosophila melanogaster*

More generally, our results suggest intriguing hypotheses about the transcriptional regulatory networks (TRNs) underlying behavior. We found that USP binds genomic regions near genes encoding several other TFs, and USP's genomic binding sites were enriched for multiple *cis*-regulatory motifs recognized by distinct classes of TFs. Together, these results suggest that many TFs could be involved in behavioral maturation. Supporting this hypothesis, our recent study demonstrated that the computationally-predicted targets of 78 TFs were enriched for genes that are differentially expressed in the context of honey bee maturation [20], and in the present study we characterized 16 of these 78 maturation-related TFs as direct targets of USP. These and other TFs were implicated in the regulation of other honey bee behavioral traits that are related to the age at onset of foraging, aspects of foraging performance and colony defense [20]. The potential involvement of such a large number of TFs underscores the complex, combinatorial nature of transcriptional networks underlying behavior. The success of both experimental and computational approaches to deduce some of the same regulatory relationships suggests that it should be possible to use a combined approach to understand comprehensively how genes and gene networks influence behavioral maturation. Future studies should test functional roles for additional TFs: understanding the role of MET and its hypothesized interactions with USP and JH will be of particular interest, and roles of predicted regulators of maturation that are not associated with JH should be tested as well.

Our results suggest that maturation-related, hormonally-regulated transcriptional networks are at least partly preserved between the periphery and the brain. Coordination between the periphery and brain is indicated by the coincident changes in behavior and fat body physiology that occur during worker maturation, and by the ability of both peripheral signals (e.g., changes in abdominal nutrient stores) and central signals (e.g., pheromones perceived as olfactory stimuli in the brain) to induce these changes [39], [73]. Our results build on previous demonstrations that JH mediates maturational changes in both the brain and periphery by showing that these diverse hormonal actions occur through overlapping transcriptional mechanisms in different tissues. Transcriptional regulation of *usp* by JH in honey bee brain has been shown previously [46]; future experiments should test the hypothesis that USP mediates transcriptional responses to JH in the brain via the same target genes that we characterized in the fat bodies. We speculate that JH and USP regulate many of the same direct target genes in both tissues, and that tissue-specific responses might result from interactions with unknown, tissue-specific transcription factors, leading to the activation of different downstream effector genes.

Transcription factors influence behavior via a variety of mechanisms over different timescales. *FoxP2* – which influences language abilities in humans – has been shown to regulate genes that are involved in brain development and that are differentially expressed in the brains of humans and chimpanzees [3], suggesting that this TF induces evolutionary, lifelong differences in behavior through its effects on the development of neuronal circuits. Other transcription factors, such as *Creb* and *Fos*, induce target gene expression in a cell-specific manner following neuronal activity [74], suggesting highly localized, acute timescale functions in the adult brain. Our results demonstrate transcription factor behavioral effects over an intermediate timescale – days to weeks – by mediating gene expression changes that occur outside the brain. The behavioral repertoires of individuals and species presumably arise from transcriptional regulatory mechanisms acting over all these timescales and cell types, together linking the genome and the environment to behavior.

## Methods

### Bees

Bees were maintained at the University of Illinois Beekeeping Facility according to standard beekeeping practices. We used exclusively bees from source colonies headed by single-drone inseminated queens to reduce within-trial genetic variation; all experiments were replicated in at least two independent trials using queens from distinct European genotypes.

### *usp* RNAi

We used previously described dsUSP probes to knock down USP expression [27]; *dsUSP* was provided as a gift from Beeologics Inc. (Rehovot, Israel). Control dsRNA was *dsGFP* (also a gift from Beelogics) or (in a few behavioral trials) dsRNA matching the pUC vector, synthesized with standard *in vitro* transcription methods using T7 RNA polymerase. One-day-old bees were injected intra-abdominally with 20 ug dsUSP or control dsRNA dissolved in 1 ul ddH2O, as described [48]. Bees were painted with an identifying mark on the thorax (Testor's enamel) and placed into Plexiglas cages containing ca. 25 bees with equal numbers from each group, fed pollen paste (45% pollen/45% honey/10% water) and sugar syrup (50% sucrose w/v in water), replaced daily (as in [56]).

### Age at onset of foraging

Experimental procedures for behavioral experiments were modified slightly from [56]. 3 days after dsRNA treatment, bees were placed into small, experimental colonies that also contained ca. 1000 1-d-old bees, a queen, and honeycomb frames containing honey and pollen. We observed the hive entrance during peak foraging times (at least 3 h/d) for the following 5 days and marked bees as they returned from their first foraging flight. To account for any differences between groups in survival and for absconding bees, we counted all the bees remaining in the hive on the evening after the last day of observations.

### ChIP–chip

We collected age-matched 8–10-day-old nurses observed placing their heads into honeycomb cells containing larvae, and 21–23-day-old foragers returning to the hive carrying nectar or pollen (these were standard behavioral assays and typical ages of bees performing these tasks [57], [75]). We dissected fat body tissue from freshly collected bees and immediately performed cross-linking reactions and isolated nuclei from fat cells pooled from 8 individual bees. Chromatin immunoprecipitation was then performed on fresh material or nuclei stored at −80°C for up to 1–2 months using the EZ-ChIP kit (Millipore, Billerica, MA) according to standard protocols and a custom antibody specific to honey bee USP (Figure S3). We used custom genomic tiling microarrays (Nimblegen, Madison, WI) with 50 bp probes and 100 bp resolution designed from the *A. mellifera* genome sequence Assembly 4.0. Each two-color array was hybridized with genomic DNA pulled down using the a-USP antibody and with input genomic DNA, and the binding intensity of USP was calculated as their ratio. Hybridization and data extraction were performed according to standard operating procedures by NimbleGen. We used the Mpeak [76] and Tamalpais algorithms to identify specific peak regions bound by USP and described the union of regions identified by these programs as putative USP binding sites.

### ChIP–qPCR

We validated a few of the USP binding sites in foragers (using an antibody that recognizes a different part of the USP protein; Figure S3) and qPCR. We selected binding sites located upstream of the *5ht7* and *abl* genes (peaks were located at *A. mellifera* Assembly 4 Linkage Group (LG) 7.14 38312–38952, and LG 14.21 26426–26967, respectively), as well as two negative control regions (LG 1.17 1–4700; LG 1.21 1000–5000). ChIP was performed using a-USP and (control) pre-immunization antisera. qPCR was performed using an ABI Prism 7900 sequence detector. Specific binding of USP at each peak region was quantified as the ratio of DNA pulled down by a-USP/pre-immunization antisera at each peak region, normalized by the average of the two negative control genomic regions. Three biological replicates were performed using fat body tissue from foragers collected from three different colonies.

### mRNA–seq analysis of *usp* RNAi and JH analog treatments in the fat bodies

Bees from the two source colonies showing the strongest behavioral responses to *usp* RNAi were treated combinatorially with *usp* RNAi and JH analog treatments in a 2×2 factorial design. The JH analog methoprene was added into food during the third day of caging at a concentration of 20 mg/g food. Bees were killed by flash freezing at the end of the third day of caging. Fat bodies and annealing cuticle were dissected away from the gut after treatment with RNAlater-ICE (Ambion). Total RNA was extracted from dissected fat body tissue using RNeasy kits (Qiagen). We confirmed knockdown of *usp* by RT-qPCR, performed as previously described [77], and we selected individuals showing typical knockdown for mRNA sequencing. mRNA libraries were constructed from 4 biological replicates per group using the Illumina (San Diego, CA) mRNA-seq protocol (June, 2010 version) with multiplex adapters. Each replicate contained pooled RNA from 4 individual bees. We synthesized 75 nt mRNA sequences using an Illumina Genome Analyzer IIx, with 4 indexed libraries in each lane, to a read depth of ca. 4–9 million reads/library. Library construction and mRNA sequencing were performed at the University of Illinois W.M. Keck Center for Comparative and Functional Genomics. Reads were mapped to the *A. mellifera* Pre-release 2 Official Gene Set using the Bowtie rapid alignment tool [78]. Reads mapping to genomic locations outside these gene models and unmapped reads were not included in analyses of differential gene expression. We also mapped reads to the *A. mellifera* genome, Assembly 4, primarily for visualization. We found sequences mapping to 10406 OGS gene models, 9323 of which had >5 reads in at least one library; this is similar to the transcript diversity quantified in this tissue using microarrays [17]. We characterized differentially expressed genes by implementing a generalized linear model and Analysis of Deviance in the DESeq package in R [79], accounting for the effects of colony, dsRNA, and JHA. Few genes were found to have a significant dsRNA x JHA interaction after accounting for variance outliers, so this term was removed from the final statistical model.

### Motif enrichment analysis of all USP bound loci

We searched for binding sites using a compendium of 602 cis-regulatory motifs compiled from FlyREG (*D. melanogaster*), TRANSFAC (*D. melanogaster*, *Homo sapiens*), Jaspar (*H. sapiens*), and from [80] (*D. melanogaster*) (essentially as in [81]). We scanned each of the 1360 USP binding loci (ChIP peaks) for one or more matches to a motif (say “*M*”), using the Patser program with default parameters. We repeated this for 1360 random genomic segments selected to match the lengths of the USP binding loci, thus obtaining a 2×2 contingency table of 1360×2 = 2720 sequences, categorized as “USP-binding” vs. “random genomic” and “*M* present” vs. “*M* absent”. A Fisher's exact test provided a p-value for this contingency table, which was used as a measure of the statistical significance of association between *M* and USP bound loci.

### Associations between motifs and specific classes of transcriptional responses

We considered the 117 USP-binding loci located near genes that are differentially regulated in foragers or in nurses, categorizing them as being “induced in foragers” as opposed to “induced in nurses”. These same loci were also categorized as “*M* present” vs. “*M* absent” (*M* being the motif), as described above, and the resulting 2×2 contingency table was subjected to a Fisher's exact test.

### Statistical test for constraints on inter-site spacing

For any two motifs M_1_ and M_2_, we used Patser to predict sites, and categorize all adjacent pairs of heterotypic sites, over all 1360 USP-bound loci, as having inter-site distance < = 25 bp or >25 bp. We artificially constructed “background” data sets where each of the 1360 original sequences had the locations of its binding sites randomly shuffled, pooled together 50 such data sets, and categorized the adjacent pairs of heterotypic sites as having inter-site distance < = 25 bp or >25 bp. We compared the counts from the original data set with the counts from the artificially constructed background sets, using a Fisher's exact test.

### Manipulations of diet quality and *vg* RNAi

We generated both fat body and brain gene expression profiles from 60 bees after manipulations of diet quality or *vg* RNAi. To manipulate diet quality, groups of 35 1-d-old bees were placed into Plexiglas cages and fed either a rich diet containing pollen paste (45% pollen/45% honey/10% water) and sugar syrup (sucrose 50% w/v in dH2O), or a poor diet containing sugar syrup only [56]. To induce *vg* RNAi, 5 ug *vg* dsRNA [48] in 1 ul saline was injected into the abdomens of 1-d-old bees, compared to bees injected with saline alone, or mock manipulated; bees were placed into Plexiglas cages and fed a rich diet. In both experiments, bees were killed by flash freezing after 3 d and stored at −80°C.

### Microarray gene expression profiling of fat bodies and whole brains

RNA was extracted from dissected fat bodies (as above) and from dissected whole brains [57] of bees from the diet quality and *vg* RNAi experiments. Sample processing, microarray procedures, and statistical analyses were essentially as in [69]; we used separate loop designs for each experiment and tissue, each replicated with a total of 10–20 bees/group. RNA from the fat bodies or brains of individual bees was subjected to one round of linear amplification and labeled with fluorescent dye (Cy3 or Cy5) using the MessageAmpII kit (Ambion) in combination with a Universal Labeling System (Kreatech). Labeled aRNA was hybridized to a custom, oligonucleotide microarray containing 28,800 oligos, including 13,440 duplicately spotted experimental probes, primarily based on gene models from the Honeybee Genome Sequencing Project. Slides were scanned with an Axon 4000B scanner, and images were analyzed with GENEPIX software (Agilent Technologies). Expression intensity data were normalized using a Loess transformation implemented in *Beehive* (http://stagbeetle.animal.uiuc.edu/Beehive). A linear mixed-effects model implemented by using restricted maximum likelihood was used to describe the normalized log_2_-transformed gene intensities values, including the effects of experimental variables, dye, bee, and microarray. Effects were evaluated with an F-test statistic and the P-values were adjusted for multiple hypothesis testing by using a FDR criterion. The effects of diet quality and *vg* RNAi on fat body gene expression were described previously [17]. For brain datasets, we removed from the resulting gene lists genes that are abundantly expressed in the hypopharyngeal glands, a potential source of contamination. Microarray data meet Minimum Information about Microarray Experiment (MIAME) standards and are available at ArrayExpress (http://www.ebi.ac.uk/microarray-as/ae/): accession numbers E-MTAB-495 (effects of maturation and diet on fat body tissue), E-MTAB-507 (effects of diet on brain), E-MTAB-490 (effects of Vg RNAi on brain).

### Gene co-expression analysis

The diet quality and *vg* RNAi experiments together included a total of 60 bees for which we profiled gene expression in both the brain and fat bodies. We generated expression estimates for these individual bees by implementing a linear mixed-effects model including the effects of dye, bee, and microarray (but not experimental variables). We then merged all the individual bee estimates from each tissue and performed a quantile normalization to create a uniform dataset suitable for gene co-expression analysis. We used the following combination of established methods to identify gene co-expression modules that were shared between fat and brain (called “coherent” modules), as well as co-expression relationships specific to the fat bodies. We first used Weighted Gene Co-expression Network Analysis (WGCNA) [67] to identify large groups of co-expressed genes in the fat bodies (each of these modules contains >30 genes). We then applied TightCluster [68] to extract smaller clusters with the most tightly co-expressed groups of genes in each of the modules generated by WGCNA. Finally, we used CoherentCluster [68] to extract subsets of genes within these fat body-based tight clusters which were also co-expressed with each other in the brain; we call the sets of genes that are co-expressed with each other in both tissues “coherent” clusters. In addition, we identified clusters of genes that were co-expressed with each other only in the fat bodies but not in the brain; these fat-specific clusters consist of the portions of tight clusters that remain after removing genes identified in coherent clusters. We examined the statistical enrichment of clusters for differentially-expressed genes responding to USP, JHA, Vg, and maturation treatments using Fisher's Exact Tests and Chi-square tests.

## Supporting Information

Figure S1 Genotypic differences in the effects of *usp* on foraging ontogeny. The effects of *usp* RNAi on the age at onset of foraging were measured using bees from 6 genotypically distinct source colonies headed by queens of several different European sub-species. In the first trial for each genotype bees from different genotypes were placed into separate single-cohort colonies. We noticed differences in the strength – and in one case the direction – of the response to *usp* RNAi. Data are shown for the two genotypes in which *usp* RNAi caused the strongest delay (Genotypes A and B), and for the genotype for which *usp* RNAi caused an acceleration of foraging ontogeny (Genotype C). We measured the foraging ontogeny of additional bees from these three genotypes in a second trial, in which bees from the three genotypes were placed together in the same experimental colony after treatment with dsRNA. Consistent with the results from the first trial, *usp* RNAi caused delayed foraging ontogeny of bees from Genotypes A and B but not from Genotype C. The graph shown in Figure 1 represents pooled data from all the trials shown here as well as from trials using 3 additional genotypes that displayed intermediate responses to *usp*. P-values are based on Cox Proportional Hazards modeling.
(PDF)

Figure S2 RT-qPCR validation for 4 *usp* RNAi responsive genes identified by RNA-seq. qPCR validation was performed with samples from one of the two colonies (N = 8 bees/group). qPCR provided statistical support (P<0.05) for 2 of 4 genes. Relative expression levels reported by qPCR and RNA-seq appear similar for all 4 genes. Note: since the validation was done with only one colony, statistical power was reduced compared to RNA-seq.
(PDF)

Figure S3 Western blots using antibodies that recognize honey bee USP. Fat body protein extracts were used in both assays. Left lane: the antibody used in ChIP-chip. Right lane: a second USP antibody generated using a different peptide antigen, which was used for validation with ChIP-qPCR. The two lanes were treated separately with the two primary antibodies but were otherwise handled together.
(PDF)

Figure S4 Validation of USP binding sites by ChIP-qPCR. To validate USP binding sites identified in ChIP-chip, we prepared additional biological samples from the fat bodies of foragers and assayed USP binding at peak regions located near the genes *5ht7* and *abl*. For these assays, ChIP was performed using an a-USP antibody that recognizes a different part of the USP protein than the antibody used in ChIP-chip (see Figure S3). Specific binding of USP in these regions was assayed by comparing the quantity of DNA pulled down by a-USP antisera to pulldowns with pre-immunization blood from the same animal, normalized to two negative control regions. DNA from both peak regions was generally more abundant in a-USP than pre-bleed ChIP pulldowns, though this enrichment was marginally insignificant (paired t-tests, n = 3: *5ht7*, *P = 0.053*; *abl*, *P = 0.098*). Though statistically inconclusive, these results support specific binding of USP in these peak regions and the results from ChIP-chip.
(PDF)

Figure S5 Expression of *E75* and *Chd64* in the fat bodies of nurses and foragers. The transcription factors *E75* and *Chd64* have previously been implicated in juvenile hormone signaling in other species. In addition, we identified both as genomic targets of USP in the bee. Microarray gene expression profiling revealed that both are expressed more highly in forager than nurse fat bodies (FDR<0.05).
(PDF)

Figure S6 RT-qPCR validation for 4 juvenile hormone analog responsive genes identified by RNA-seq. qPCR validation was performed with samples from one of two colonies (N = 8 bees/group). qPCR confirmed significant responses to JHA (P<0.05) for 4 of 4 genes.
(PDF)

Figure S7 Validation of juvenile hormone effects on behavioral maturation. Many previous experiments have demonstrated causal roles for juvenile hormone (JH) in behavioral maturation. In this study, we administered JH analog treatments (20 mg JHA) to 3-d-old bees rather than 1-d-old bees in order to facilitate comparisons with *usp* RNAi (which took effect when bees were 3-d-old). We confirmed that JH analog treatments had similar effects on behavioral maturation when administered to either 3-d-old or 1-d-old bees. Chi-squared test on total proportion of bees foraging vs. not foraging: 20 mg JHA, 3-d-old vs. 0 mg JHA: *P = 0.002*; 20 mg JHA, 1-d-old vs. 0 mg JHA: *P = 5.4e-4*; 20 mg JHA, 3-d-old vs. 20 mg JHA, 1-d-old: *P = 0.77*. *N = 66–92* bees.
(PDF)

Figure S8 USP Mediates Transcriptional Responses to JHA. We assessed the extent to which USP is required for transcriptional responses to JHA by examining the 33 genes that were activated both by JHA and by USP (i.e., downregulated by *usp* RNAi). The boxplot shows that most of these genes responded less strongly to JHA when we knocked down USP. This was not due to a systematic bias in the dataset because we found no such pattern for other genes.
(PDF)

Figure S9 Binding strength of USP at peak regions is correlated between nurse and forager fat bodies. ChIP-chip was performed using three independent samples of fat body tissue from nurses (N1–N3) and foragers (F1–F3). USP binding intensity at each probe on the genomic tiling microarray (log-ratio of USP pulldown vs. input control) is plotted for each pair of samples.

Figure S10 Maturation and hormonal and nutritional treatments induce correlated transcriptional responses in brain and fat. A) Transcriptome responses were measured in the fat bodies and whole brains for maturation (forager – nurse), juvenile hormone analog (JHA) treatments, vitellogenin (Vg) RNAi, and diet (nutrient-poor sugar-only diet – nutrient-rich pollen/honey/sugar diet). Scatterplots show log-transformed fold changes in each tissue for the set of genes that were differentially expressed in both tissues. For each experiment, we report the number of genes in this set and a P-value for enrichment (hypergeometric tests), as well as the coefficient and P-value for the (Pearson) correlation between fold change responses of these genes in the two tissues. B) The analysis shown in (A) had considerably less power for JHA than for the other experiments because transcriptome measurements were made on different platforms (brain: cDNA microarray with ca. 6000 features; fat: digital gene expression based on mRNA-sequencing) that allowed direct comparison of only 3094 genes (ca. 25%). To increase statistical power we examined the brain vs. fat fold change correlations for the broader sets of genes that were differentially expressed (DE) in each of the two tissues. We report the number of genes analyzed and the strength of the between-tissue correlation between fold changes.
(PDF)

Figure S11 Behavioral maturation and hormonal and nutritional factors induce concordant gene expression changes in the brain and fat bodies. Genes are shown that respond in the same direction in the two tissues, for at least 2 experiments. In each experiment, one condition corresponds to “fast” maturation and the other to “slow” maturation. Blue: gene is significantly higher in the “fast” condition (i.e., forager>nurse, poor diet>rich diet; vg RNAi>control; JHA>control). Yellow: gene is higher in the slow condition. Black: gene is not significantly differentially expressed. Gray: no data. Stars indicate genes that have been linked previously to juvenile hormone signaling (manual annotation). Genes are named according to the symbol of their *D. melanogaster* ortholog or (for genes without a clear ortholog) by their *A. mellifera* Official Gene Set symbol. Average-linkage hierarchical clustering is used primarily for visualization purposes.
(PDF)

Table S1 Genes differentially expressed in fat bodies after *usp* RNAi. Gene expression was profiled using mRNA-seq and differential expression was assessed with DESeq, as described in Methods. For each differentially expressed gene (False Discovery Rate<0.1) we list its *Drosophila melanogaster* ortholog (if applicable); the FDR-adjusted p-values for response to USP RNAi, juvenile hormone analog (JHA) treatment, and for their interaction; log2-transformed fold changes for response to *usp* RNAi and JHA; whether each gene was previously reported to be up- or down-regulated during behavioral maturation; and whether each gene is located near a USP binding site identified by ChIP-chip.
(DOC)

Table S2 USP binding sites characterized by ChIP-chip. For each binding site, we give the chromosomal (group) location, and the locations of any genes located with within 10 kb.
(XLS)

Table S3 Genes differentially expressed in fat bodies following Juvenile Hormone Analog treatment. Gene expression was profiled using mRNA-seq and differential expression was assessed with DESeq, as described in Methods. For each differentially expressed gene (False Discovery Rate<0.1) we list its *Drosophila melanogaster* ortholog (if any); the FDR-adjusted p-values for response to USP RNAi, juvenile hormone analog (JHA) treatment, and for their interaction; log2-transformed fold changes for response to *usp* RNAi and JHA; whether each gene was previously reported to be up- or down-regulated during behavioral maturation; and whether each gene is located near a USP binding site identified by ChIP-chip.
(DOC)

Table S4 Motif associations with USP target genes that are differentially regulated in foragers or in nurses. Associations were tested using Fisher's exact test, as described in Methods. The 2×2 contingency table is shown in columns “F-motifyes” (Forager gene, motif present), “N-motifyes” (Nurse gene, motif present), “F-motifno” (Forager gene, motif absent) and “N-motifno” (Nurse gene, motif absent). The p-value is shown in column “Fisher-pval”, with its direction (overrepresented in forager genes or in nurse genes) shown in column “For/Nurse?”.
(XLS)

Table S5 Clusters of genes co-expressed in both brain and fat, and their enrichment for maturation and hormonally-related genes. Clustering was performed using a combination of Weighted Gene Co-expression Network Analysis and CoherentCluster, as described in Methods. For each cluster, we provide the identifiers for the associated microarray features; and statistical enrichments for genes that were differentially expressed in response to maturation, associated with USP (ChIP-chip or *usp* RNAi transcriptomics), and responsive to juvenile hormone analog treatment, to *vitellogenin* RNAi, or rich vs. poor diet. Note: to compare RNA-seq data with microarray data, we used the microarray features corresponding to each differentially expressed transcript.
(XLS)
