# Supplementary figures and images for: The Transcription Factor Ultraspiracle Influences Honey Bee Social Behavior and Behavior-Related Gene Expression
Source: PLoS Genet. 2012 Mar 29;8(3):e1002596. doi: 10.1371/journal.pgen.1002596 (PMC3315457; doi:10.1371/journal.pgen.1002596)

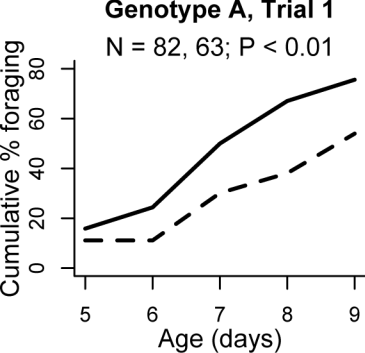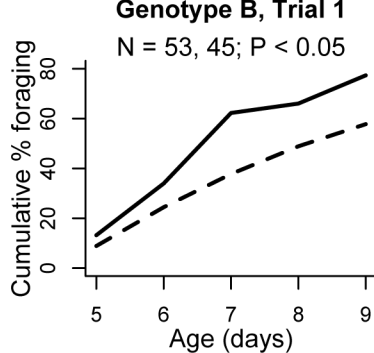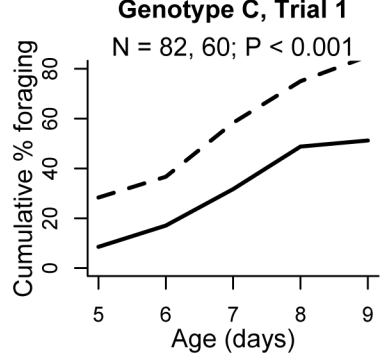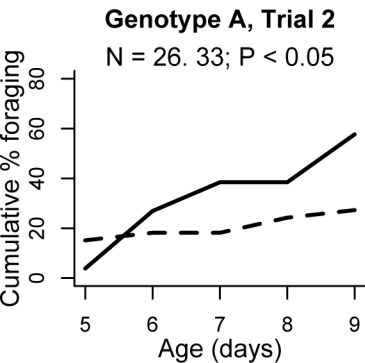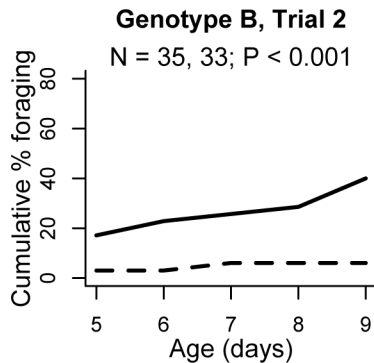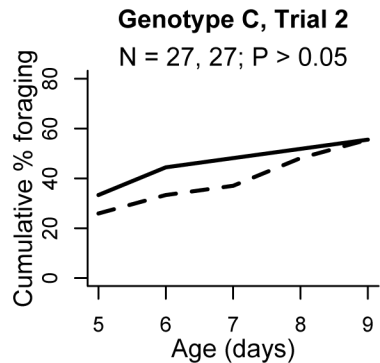

Supplement: Figure S1 — Genotypic differences in the effects of usp on foraging ontogeny. The effects of usp RNAi on the age at onset of foraging were measured using bees from 6 genotypically distinct source colonies headed by queens of several different European sub-species. In the first trial for each genotype bees from different genotypes were placed into separate single-cohort colonies. We noticed differences in the strength – and in one case the direction – of the response to usp RNAi. Data are shown for the two genotypes in which usp RNAi caused the strongest delay (Genotypes A and B), and for the genotype for which usp RNAi caused an acceleration of foraging ontogeny (Genotype C). We measured the foraging ontogeny of additional bees from these three genotypes in a second trial, in which bees from the three genotypes were placed together in the same experimental colony after treatment with dsRNA. Consistent with the results from the first trial, usp RNAi caused delayed foraging ontogeny of bees from Genotypes A and B but not from Genotype C. The graph shown in Figure 1 represents pooled data from all the trials shown here as well as from trials using 3 additional genotypes that displayed intermediate responses to usp. P-values are based on Cox Proportional Hazards modeling. (PDF) [file pgen.1002596.s001.pdf]

207 kD

114

78

53

35

28

19

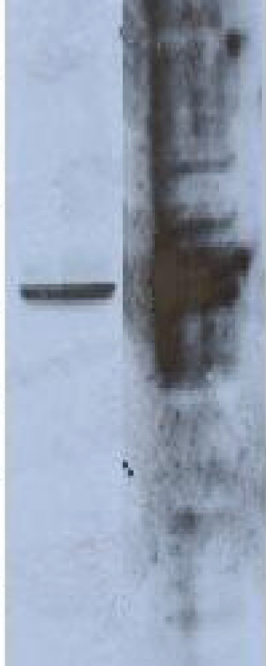

Supplement: Figure S3 — Western blots using antibodies that recognize honey bee USP. Fat body protein extracts were used in both assays. Left lane: the antibody used in ChIP-chip. Right lane: a second USP antibody generated using a different peptide antigen, which was used for validation with ChIP-qPCR. The two lanes were treated separately with the two primary antibodies but were otherwise handled together. (PDF) [file pgen.1002596.s003.pdf]

**5ht7**  
 $P = 0.053$

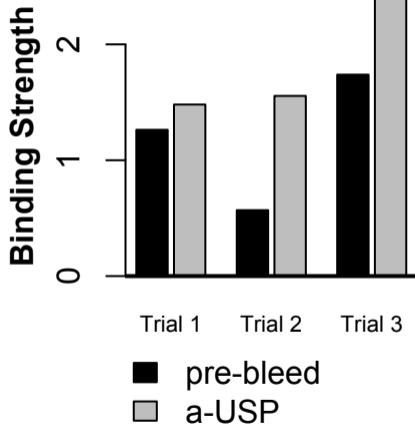

**abl**  
 $P = 0.098$

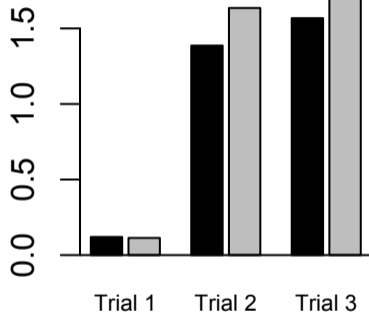

Supplement: Figure S4 — Validation of USP binding sites by ChIP-qPCR. To validate USP binding sites identified in ChIP-chip, we prepared additional biological samples from the fat bodies of foragers and assayed USP binding at peak regions located near the genes 5ht7 and abl. For these assays, ChIP was performed using an a-USP antibody that recognizes a different part of the USP protein than the antibody used in ChIP-chip (see Figure S3). Specific binding of USP in these regions was assayed by comparing the quantity of DNA pulled down by a-USP antisera to pulldowns with pre-immunization blood from the same animal, normalized to two negative control regions. DNA from both peak regions was generally more abundant in a-USP than pre-bleed ChIP pulldowns, though this enrichment was marginally insignificant (paired t-tests, n = 3: 5ht7, P = 0.053; abl, P = 0.098). Though statistically inconclusive, these results support specific binding of USP in these peak regions and the results from ChIP-chip. (PDF) [file pgen.1002596.s004.pdf]

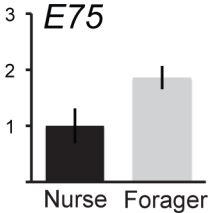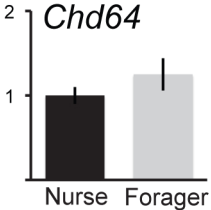

Supplement: Figure S5 — Expression of E75 and Chd64 in the fat bodies of nurses and foragers. The transcription factors E75 and Chd64 have previously been implicated in juvenile hormone signaling in other species. In addition, we identified both as genomic targets of USP in the bee. Microarray gene expression profiling revealed that both are expressed more highly in forager than nurse fat bodies (FDR<0.05). (PDF) [file pgen.1002596.s005.pdf]

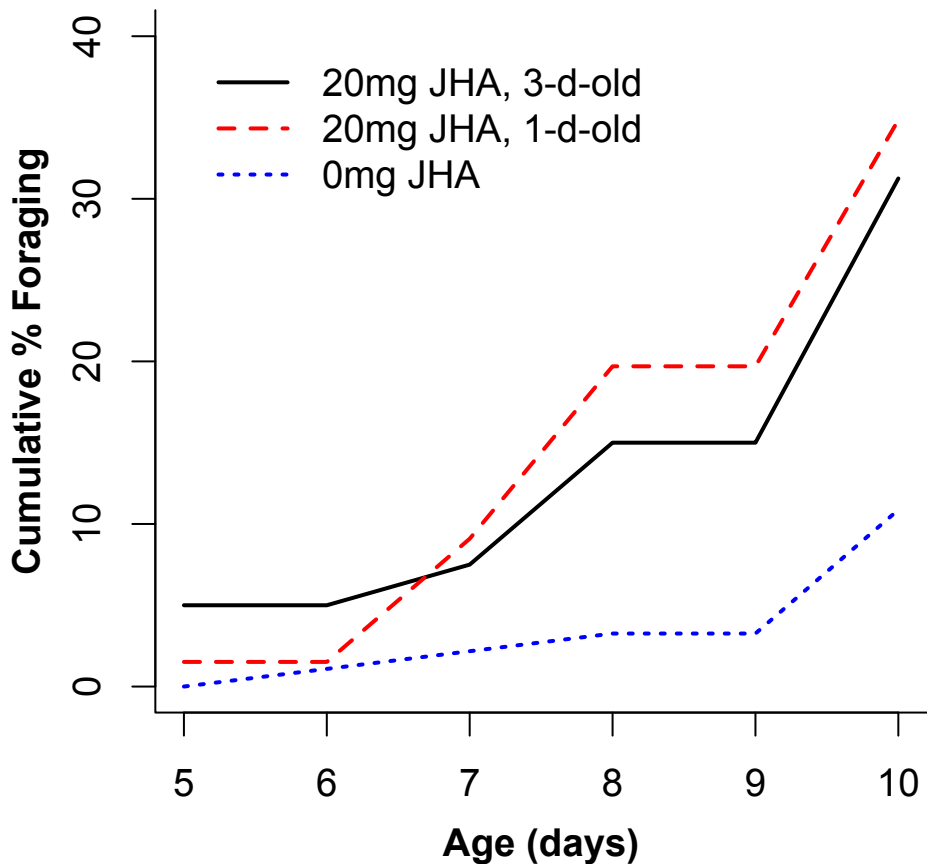

Supplement: Figure S7 — Validation of juvenile hormone effects on behavioral maturation. Many previous experiments have demonstrated causal roles for juvenile hormone (JH) in behavioral maturation. In this study, we administered JH analog treatments (20 mg JHA) to 3-d-old bees rather than 1-d-old bees in order to facilitate comparisons with usp RNAi (which took effect when bees were 3-d-old). We confirmed that JH analog treatments had similar effects on behavioral maturation when administered to either 3-d-old or 1-d-old bees. Chi-squared test on total proportion of bees foraging vs. not foraging: 20 mg JHA, 3-d-old vs. 0 mg JHA: P = 0.002; 20 mg JHA, 1-d-old vs. 0 mg JHA: P = 5.4e-4; 20 mg JHA, 3-d-old vs. 20 mg JHA, 1-d-old: P = 0.77. N = 66–92 bees. (PDF) [file pgen.1002596.s007.pdf]

**Difference in Response to JHA**

$\log_2 ( \text{JHA in dsUSP} - \text{JHA in dsGFP} )$

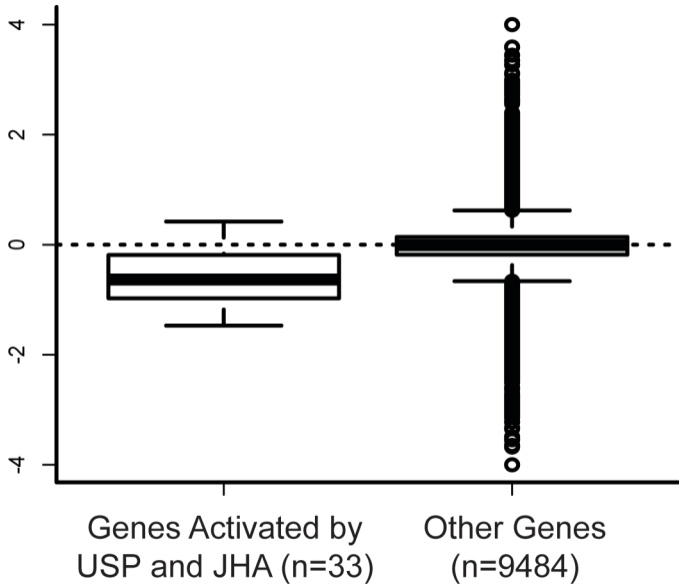

Supplement: Figure S8 — USP Mediates Transcriptional Responses to JHA. We assessed the extent to which USP is required for transcriptional responses to JHA by examining the 33 genes that were activated both by JHA and by USP (i.e., downregulated by usp RNAi). The boxplot shows that most of these genes responded less strongly to JHA when we knocked down USP. This was not due to a systematic bias in the dataset because we found no such pattern for other genes. (PDF) [file pgen.1002596.s008.pdf]

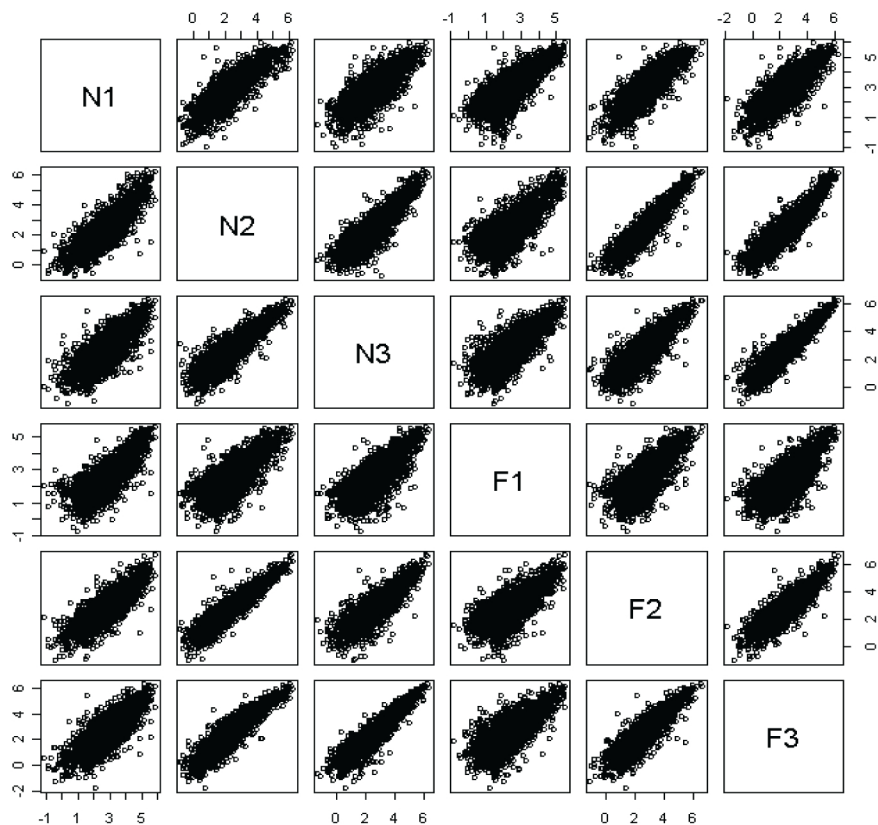

Supplement: Figure S9 — Binding strength of USP at peak regions is correlated between nurse and forager fat bodies. ChIP-chip was performed using three independent samples of fat body tissue from nurses (N1–N3) and foragers (F1–F3). USP binding intensity at each probe on the genomic tiling microarray (log-ratio of USP pulldown vs. input control) is plotted for each pair of samples. [file pgen.1002596.s009.pdf]

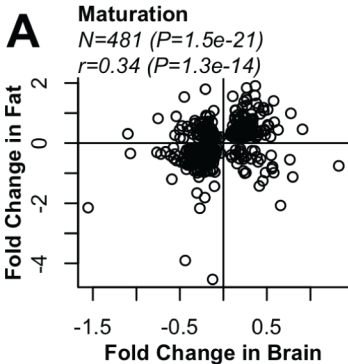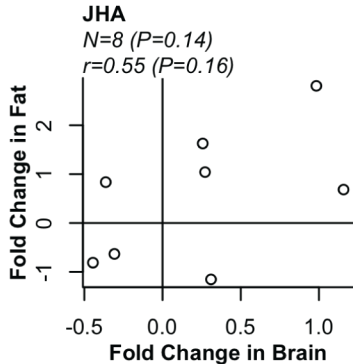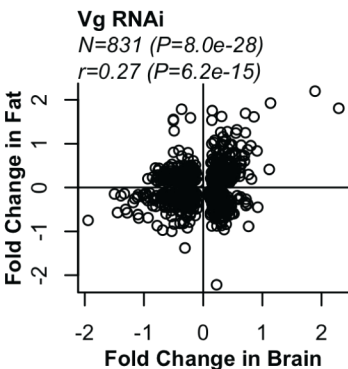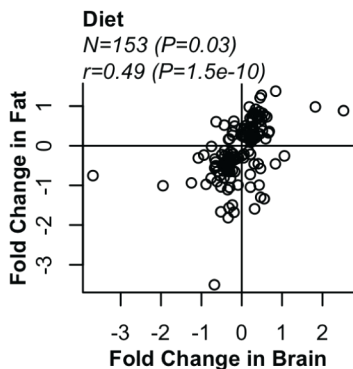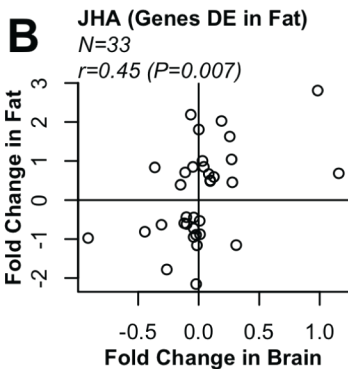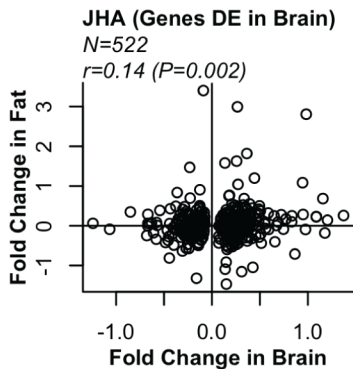

Supplement: Figure S10 — Maturation and hormonal and nutritional treatments induce correlated transcriptional responses in brain and fat. A) Transcriptome responses were measured in the fat bodies and whole brains for maturation (forager – nurse), juvenile hormone analog (JHA) treatments, vitellogenin (Vg) RNAi, and diet (nutrient-poor sugar-only diet – nutrient-rich pollen/honey/sugar diet). Scatterplots show log-transformed fold changes in each tissue for the set of genes that were differentially expressed in both tissues. For each experiment, we report the number of genes in this set and a P-value for enrichment (hypergeometric tests), as well as the coefficient and P-value for the (Pearson) correlation between fold change responses of these genes in the two tissues. B) The analysis shown in (A) had considerably less power for JHA than for the other experiments because transcriptome measurements were made on different platforms (brain: cDNA microarray with ca. 6000 features; fat: digital gene expression based on mRNA-sequencing) that allowed direct comparison of only 3094 genes (ca. 25%). To increase statistical power we examined the brain vs. fat fold change correlations for the broader sets of genes that were differentially expressed (DE) in each of the two tissues. We report the number of genes analyzed and the strength of the between-tissue correlation between fold changes. (PDF) [file pgen.1002596.s010.pdf]

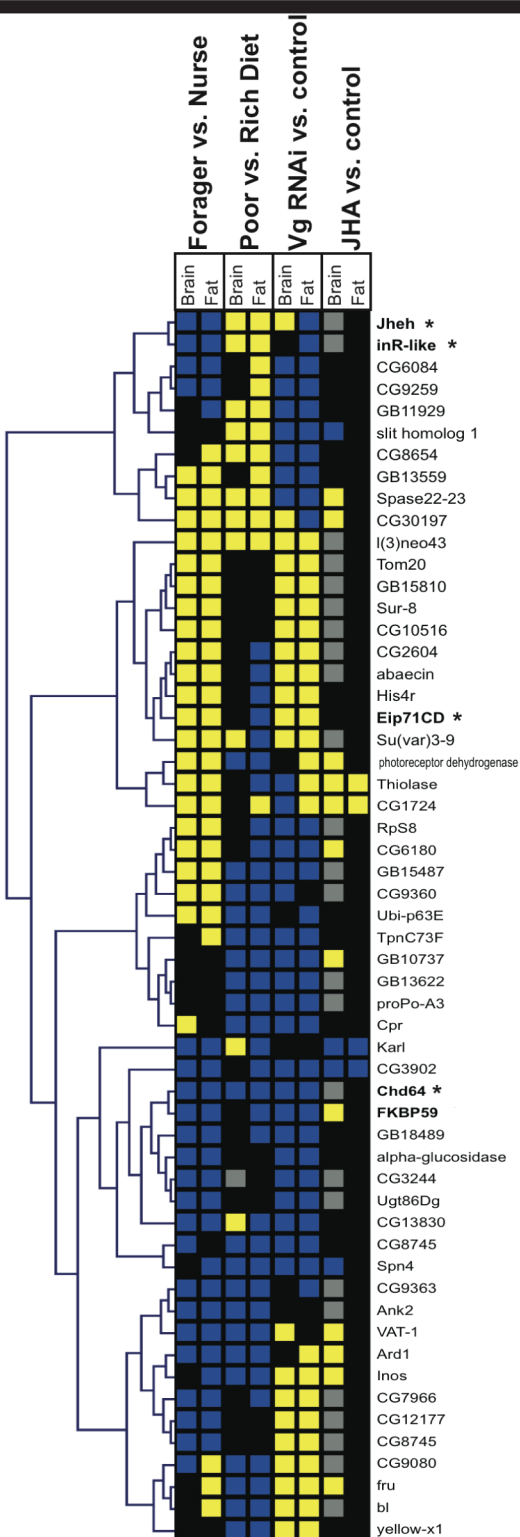

Supplement: Figure S11 — Behavioral maturation and hormonal and nutritional factors induce concordant gene expression changes in the brain and fat bodies. Genes are shown that respond in the same direction in the two tissues, for at least 2 experiments. In each experiment, one condition corresponds to “fast” maturation and the other to “slow” maturation. Blue: gene is significantly higher in the “fast” condition (i.e., forager>nurse, poor diet>rich diet; vg RNAi>control; JHA>control). Yellow: gene is higher in the slow condition. Black: gene is not significantly differentially expressed. Gray: no data. Stars indicate genes that have been linked previously to juvenile hormone signaling (manual annotation). Genes are named according to the symbol of their D. melanogaster ortholog or (for genes without a clear ortholog) by their A. mellifera Official Gene Set symbol. Average-linkage hierarchical clustering is used primarily for visualization purposes. (PDF) [file pgen.1002596.s011.pdf]
