# Supplementary material for: The Transcription Factor Ultraspiracle Influences Honey Bee Social Behavior and Behavior-Related Gene Expression
Source: PLoS Genet. 2012 Mar 29;8(3):e1002596. doi: 10.1371/journal.pgen.1002596 (PMC3315457; doi:10.1371/journal.pgen.1002596)
Supplement: Figure S2 — RT-qPCR validation for 4 usp RNAi responsive genes identified by RNA-seq. qPCR validation was performed with samples from one of the two colonies (N = 8 bees/group). qPCR provided statistical support (P<0.05) for 2 of 4 genes. Relative expression levels reported by qPCR and RNA-seq appear similar for all 4 genes. Note: since the validation was done with only one colony, statistical power was reduced compared to RNA-seq. (PDF) [file pgen.1002596.s002.pdf]

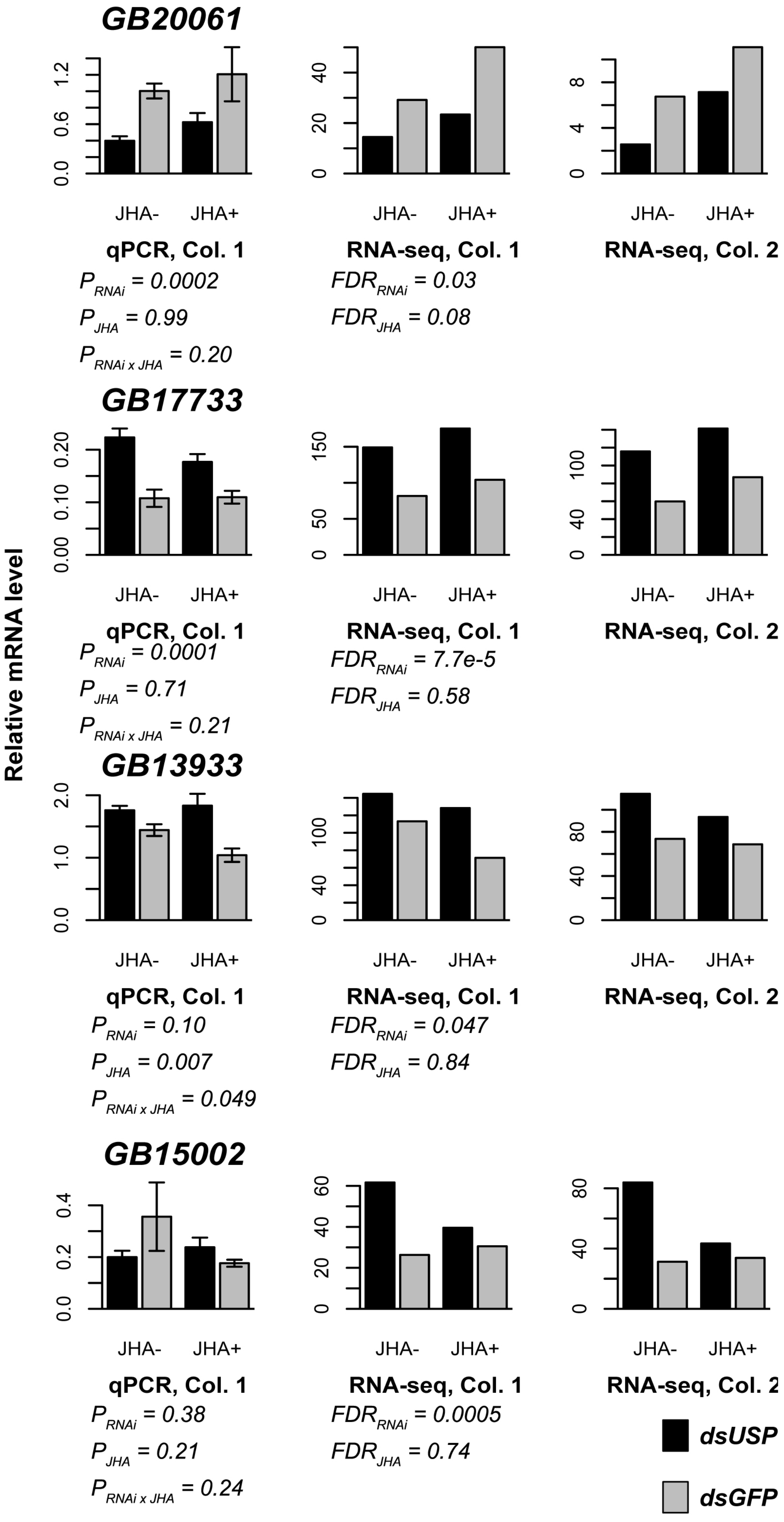
Relative mRNA level
GB20061
qPCR, Col. 1
RNA-seq, Col. 1
RNA-seq, Col. 2
JHA-
JHA+
P RNAi = 0.0002
P JHA = 0.99
P RNAi x JHA = 0.20
FDR RNAi = 0.03
FDR JHA = 0.08
GB17733
qPCR, Col. 1
RNA-seq, Col. 1
RNA-seq, Col. 2
P RNAi = 0.0001
P JHA = 0.71
P RNAi x JHA = 0.21
FDR RNAi = 7.7e-5
FDR JHA = 0.58
GB13933
qPCR, Col. 1
RNA-seq, Col. 1
RNA-seq, Col. 2
P RNAi = 0.10
P JHA = 0.007
P RNAi x JHA = 0.049
FDR RNAi = 0.047
FDR JHA = 0.84
GB15002
qPCR, Col. 1
RNA-seq, Col. 1
RNA-seq, Col. 2
P RNAi = 0.38
P JHA = 0.21
P RNAi x JHA = 0.24
FDR RNAi = 0.0005
FDR JHA = 0.74
dsUSP
dsGFP
