# Supplementary material for: The Transcription Factor Ultraspiracle Influences Honey Bee Social Behavior and Behavior-Related Gene Expression
Source: PLoS Genet. 2012 Mar 29;8(3):e1002596. doi: 10.1371/journal.pgen.1002596 (PMC3315457; doi:10.1371/journal.pgen.1002596)
Supplement: Figure S6 — RT-qPCR validation for 4 juvenile hormone analog responsive genes identified by RNA-seq. qPCR validation was performed with samples from one of two colonies (N = 8 bees/group). qPCR confirmed significant responses to JHA (P<0.05) for 4 of 4 genes. (PDF) [file pgen.1002596.s006.pdf]

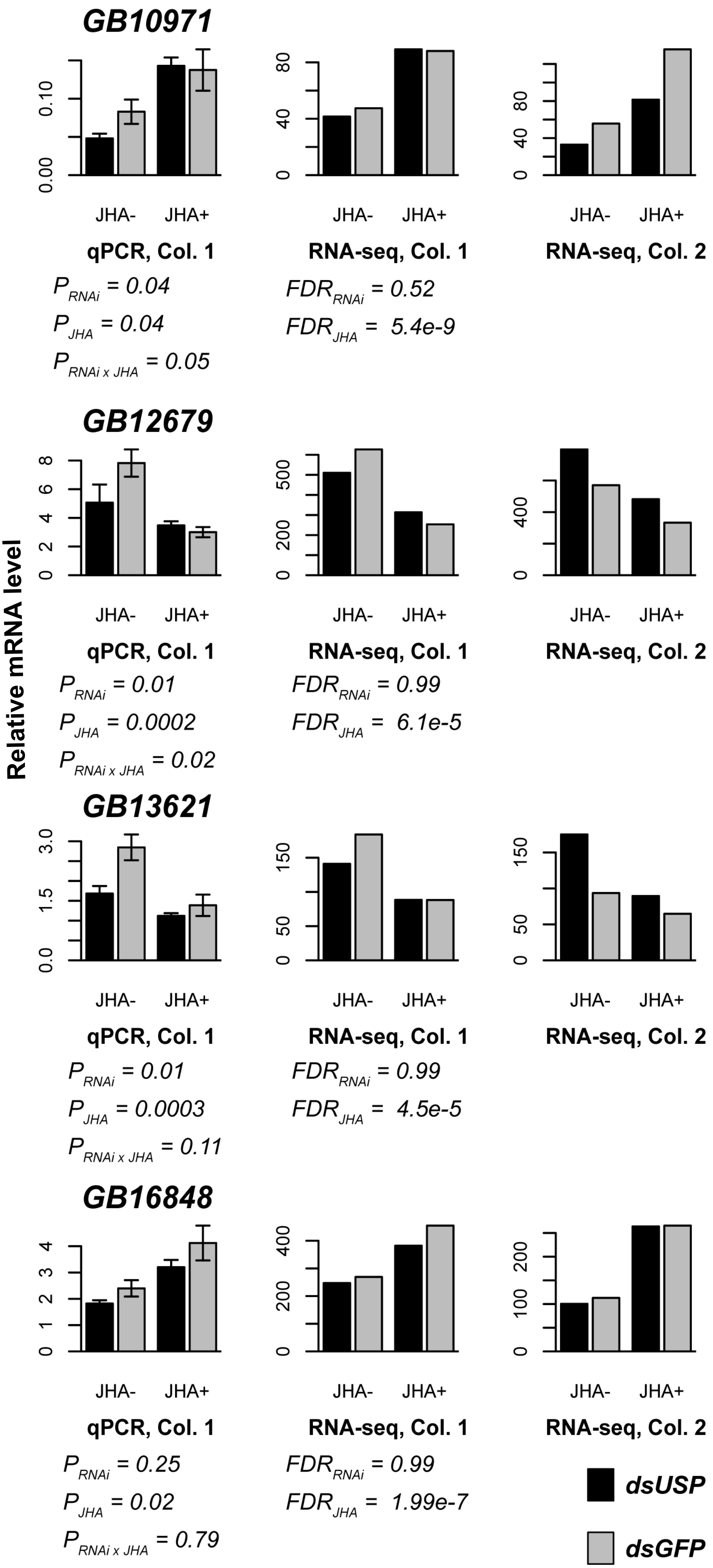

Relative mRNA level
GB10971
0.00
0.10
JHA-
JHA+
qPCR, Col. 1
P_{RNAi} = 0.04
P_{JHA} = 0.04
$P_{RNAi \times JHA}$ = 0.05
0
40
80
RNA-seq, Col. 1
FDR_{RNAi} = 0.52
FDR_{JHA} = 5.4e-9
0
40
80
RNA-seq, Col. 2
GB12679
0
2
4
6
8
qPCR, Col. 1
P_{RNAi} = 0.01
P_{JHA} = 0.0002
$P_{RNAi \times JHA}$ = 0.02
0
200
500
RNA-seq, Col. 1
FDR_{RNAi} = 0.99
FDR_{JHA} = 6.1e-5
0
400
RNA-seq, Col. 2
GB13621
0.0
1.5
3.0
qPCR, Col. 1
P_{RNAi} = 0.01
P_{JHA} = 0.0003
$P_{RNAi \times JHA}$ = 0.11
0
50
150
RNA-seq, Col. 1
FDR_{RNAi} = 0.99
FDR_{JHA} = 4.5e-5
0
50
150
RNA-seq, Col. 2
GB16848
0
1
2
3
4
qPCR, Col. 1
P_{RNAi} = 0.25
P_{JHA} = 0.02
$P_{RNAi \times JHA}$ = 0.79
0
200
400
RNA-seq, Col. 1
FDR_{RNAi} = 0.99
FDR_{JHA} = 1.99e-7
0
100
200
RNA-seq, Col. 2
dsUSP
dsGFP
