# Supplementary material for: The Transcription Factor Ultraspiracle Influences Honey Bee Social Behavior and Behavior-Related Gene Expression
Source: PLoS Genet. 2012 Mar 29;8(3):e1002596. doi: 10.1371/journal.pgen.1002596 (PMC3315457; doi:10.1371/journal.pgen.1002596)
Supplement: Table S1 — Genes differentially expressed in fat bodies after usp RNAi. Gene expression was profiled using mRNA-seq and differential expression was assessed with DESeq, as described in Methods. For each differentially expressed gene (False Discovery Rate<0.1) we list its Drosophila melanogaster ortholog (if applicable); the FDR-adjusted p-values for response to USP RNAi, juvenile hormone analog (JHA) treatment, and for their interaction; log2-transformed fold changes for response to usp RNAi and JHA; whether each gene was previously reported to be up- or down-regulated during behavioral maturation; and whether each gene is located near a USP binding site identified by ChIP-chip. (DOC) [file pgen.1002596.s012.doc]

**Table S1. Genes differentially expressed in fat bodies after *usp* RNAi.**

| **ID** | **D.mel. Ortholog** | **p.adj(JHA)** | **p.adj(USP)** | **p.adj(JHAxUSP)** | **log2(JHA-Control)** | **log2(dsGFP-dsUSP)** | **Maturation** | **ChIP** |
| --- | --- | --- | --- | --- | --- | --- | --- | --- |
| *GB10128* | *CG5130* | 8.80E-01 | 5.79E-03 | 1 | 0.53 | 1.01 |  |  |
| *GB10147* | *Dhc93AB* | 1.00E+00 | 1.27E-07 | 1 | 0.05 | 4.16 |  |  |
| *GB10252* | *CG6597* | 9.42E-02 | 4.48E-02 | 1 | 0.74 | 0.74 |  |  |
| *GB10428* | *CG33998* | 1.73E-12 | 7.89E-06 | 1 | 2.01 | 1.70 | Forager High |  |
| *GB10764* |  | 4.22E-02 | 9.05E-03 | 1 | -1.55 | 2.08 |  |  |
| *GB10910* |  | 1.00E+00 | 4.73E-02 | 1 | 0.20 | 0.54 | Forager High |  |
| *GB11007* | *Set* | 1.00E+00 | 9.02E-02 | 1 | 0.16 | 0.73 |  |  |
| *GB11111* |  | 8.28E-03 | 2.04E-02 | 1 | 1.81 | 1.48 |  |  |
| *GB11152* | *CG3332* | 1.00E+00 | 9.61E-02 | 0.60 | 0.23 | 1.23 |  |  |
| *GB11256* |  | 1.04E-07 | 7.93E-05 | 1 | -1.29 | 1.04 |  |  |
| *GB11499* | *CG31004* | 1.44E-01 | 6.53E-06 | 1 | 0.91 | 1.77 |  |  |
| *GB11581* | *Adgf-A* | 2.46E-03 | 2.19E-03 | 1 | 1.02 | 0.92 |  |  |
| *GB12136* |  | 1.60E-02 | 1.45E-03 | 1 | -4.22 | Inf |  |  |
| *GB12136* |  | 1.60E-02 | 1.45E-03 | 1 | -4.22 | Inf |  |  |
| *GB12158* |  | 2.36E-02 | 1.62E-04 | 1 | 0.11 | 1.26 | Nurse High |  |
| *GB12203* |  | 6.58E-11 | 2.17E-04 | 1 | 2.69 | 1.32 | Forager High |  |
| *GB12206* |  | 1.00E+00 | 2.53E-02 | 1 | -0.34 | 1.55 | Forager High |  |
| *GB12226* | *RNaseX25* | 7.76E-01 | 6.74E-02 | 1 | 0.38 | 0.73 |  |  |
| *GB12259* | *CG42249* | 9.55E-02 | 1.18E-03 | 1 | 0.74 | 1.16 |  |  |
| *GB12292* |  | 4.78E-02 | 7.98E-02 | 1 | -1.58 | 1.77 | Nurse High |  |
| *GB12340* |  | 0.00E+00 | 1.75E-03 | 1 | -1.38 | -0.62 | Nurse High |  |
| *GB12353* |  | 2.77E-06 | 1.91E-02 | 1 | 2.45 | 1.42 | Forager High |  |
| *GB12511* |  | 4.29E-01 | 2.94E-03 | 1 | 0.78 | 0.90 |  |  |
| *GB12518* | *bowl* | 8.45E-01 | 5.66E-02 | 1 | 1.92 | 3.65 |  |  |
| *GB12607* |  | 1.29E-06 | 5.50E-03 | 1 | 2.81 | 1.22 | Forager High |  |
| *GB12716* |  | 1.00E+00 | 3.74E-03 | 1 | -1.56 | 2.23 | Nurse High |  |
| *GB12745* | *CG13196* | 4.36E-08 | 4.31E-02 | 1 | -1.87 | -1.02 |  |  |
| *GB12896* |  | 3.62E-03 | 1.71E-05 | 1 | 0.97 | 1.36 | Forager High |  |
| *GB13098* | *CG4334* | 1.00E+00 | 5.30E-02 | 1 | 0.37 | 0.94 |  |  |
| *GB13257* |  | 1.00E+00 | 9.00E-02 | 1 | -0.18 | 1.23 |  |  |
| *GB13501* |  | 1.00E+00 | 3.76E-02 | 1 | 1.17 | 1.37 |  |  |
| *GB13546* |  | 4.78E-02 | 1.49E-04 | 1 | 2.53 | 1.29 |  |  |
| *GB13620* | *CG15533* | 1.43E-02 | 2.61E-03 | 1 | 1.34 | 1.62 |  |  |
| *GB13845* | *CG14431* | 1.00E+00 | 7.41E-02 | 1 | -0.53 | 0.73 | Nurse High |  |
| *GB13933* | *CG12880* | 8.40E-01 | 4.65E-02 | 1 | -0.30 | -0.56 |  |  |
| *GB13938* | *lush* | 1.00E+00 | 9.80E-02 | 1 | 0.30 | Inf |  |  |
| *GB14169* |  | 4.60E-07 | 8.50E-04 | 1 | 1.52 | 1.28 |  |  |
| *GB14190* |  | 1.00E+00 | 2.19E-03 | 1 | 1.76 | Inf |  |  |
| *GB14261* | *Npc2d* | 1.67E-02 | 1.08E-11 | 0.43 | 0.66 | 1.68 | Nurse High |  |
| *GB14492* | *CG5549* | 1.00E+00 | 6.13E-05 | 1 | 0.31 | 2.26 |  |  |
| *GB14603* | *CG16996* | 3.08E-04 | 1.12E-07 | 0.74 | 1.52 | 1.53 | Nurse High |  |
| *GB14609* |  | 1.00E+00 | 6.44E-03 | 1 | 0.53 | 2.37 |  |  |
| *GB14784* | *CG15712* | 4.52E-06 | 9.03E-08 | 0.87 | 1.20 | 1.27 | Nurse High | Yes |
| *GB14905* |  | 1.00E+00 | 6.51E-02 | 1 | -0.08 | -0.68 |  |  |
| *GB15002* | *CG42366* | 7.43E-01 | 4.84E-04 | 1 | -0.46 | -0.91 |  |  |
| *GB15176* | *CG17029* | 5.53E-01 | 5.42E-02 | 1 | 0.34 | 0.99 |  |  |
| *GB15220* |  | 1.00E+00 | 8.73E-02 | 1 | 0.13 | 0.70 |  |  |
| *GB15246* | *CG13842* | 6.15E-02 | 9.82E-02 | 1 | 2.03 | 1.30 |  |  |
| *GB15371* |  | 3.64E-01 | 1.16E-06 | 7.11E-04 | 0.93 | 1.03 | Forager High |  |
| *GB15518* | *CG9380* | 2.04E-06 | 3.91E-04 | 1 | 1.14 | 0.98 |  |  |
| *GB15746* | *CG7910* | 1.63E-01 | 7.20E-04 | 1 | -0.53 | -0.88 | Forager High |  |
| *GB15990* |  | 1.00E+00 | 3.36E-02 | 1 | 0.10 | 1.02 | Nurse High |  |
| *GB16076* |  | 6.70E-01 | 2.62E-02 | 1 | -2.20 | Inf |  |  |
| *GB16297* |  | 1.94E-01 | 9.72E-03 | 1 | -0.83 | 1.37 | Forager High |  |
| *GB16399* |  | 4.13E-07 | 1.25E-05 | 1 | 1.59 | 1.57 | Forager High |  |
| *GB16536* |  | 2.71E-02 | 4.82E-02 | 1 | 0.72 | -0.67 |  |  |
| *GB16732* |  | 1.05E-05 | 2.31E-05 | 1 | 2.04 | 1.59 |  | Yes |
| *GB16769* |  | 6.25E-05 | 7.30E-04 | 1 | 2.19 | 1.87 |  |  |
| *GB16787* | *CG30502* | 1.00E+00 | 9.43E-02 | 0.63 | 0.77 | 1.55 |  |  |
| *GB17011* |  | 1.19E-02 | 4.82E-02 | 1 | 1.20 | 1.20 |  |  |
| *GB17017* | *Oatp33Eb* | 3.49E-08 | 8.49E-08 | 0.51 | 1.56 | 1.49 |  |  |
| *GB17068* |  | 1.00E+00 | 5.13E-04 | 1 | 0.17 | -0.63 |  |  |
| *GB17166* | *MESR3* | 1.17E-07 | 7.60E-04 | 1 | 2.19 | 1.66 |  |  |
| *GB17253* | *kkv* | 5.04E-01 | 3.36E-02 | 1 | 0.54 | 0.82 |  |  |
| *GB17481* | *CG11626* | 1.78E-05 | 2.65E-07 | 0.09 | 1.18 | 1.34 |  |  |
| *GB17733* | *Cht3* | 5.86E-01 | 7.73E-05 | 1 | 0.32 | -0.81 |  |  |
| *GB17784* | *Cyp4aa1* | 8.40E-01 | 9.43E-02 | 0.65 | -0.78 | -0.29 |  |  |
| *GB17987* | *CG7627* | 2.62E-01 | 5.13E-03 | 1 | 0.61 | 1.00 |  | Yes |
| *GB18161* |  | 1.46E-08 | 2.12E-04 | 1 | -1.83 | 1.42 | Nurse High |  |
| *GB18264* |  | 2.27E-02 | 1.45E-04 | 1 | 1.25 | 1.83 | Forager High |  |
| *GB18334* |  | 0.00E+00 | 3.91E-08 | 0.50 | 2.38 | 1.10 | Forager High |  |
| *GB18360* | *CG11796* | 2.32E-02 | 9.21E-03 | 1 | 0.87 | 0.85 | Forager High |  |
| *GB18947* |  | 9.45E-03 | 1.45E-03 | 1 | 0.86 | 0.96 |  | Yes |
| *GB19006* | *CG3884* | 7.72E-04 | 9.61E-02 | 1 | 1.13 | 0.95 | Forager High |  |
| *GB19006* | *CG3884* | 3.17E-08 | 9.05E-03 | 1 | 2.05 | 1.01 | Forager High |  |
| *GB19099* |  | 4.23E-08 | 4.87E-05 | 1 | 1.74 | 1.42 |  |  |
| *GB19302* |  | 1.00E+00 | 4.90E-02 | 1 | -0.89 | 2.54 |  |  |
| *GB19401* |  | 6.48E-05 | 3.58E-04 | 1 | 2.49 | 2.20 |  |  |
| *GB19643* |  | 9.92E-04 | 4.76E-02 | 1 | 0.94 | 0.98 |  |  |
| *GB19761* | *CG30503* | 1.00E+00 | 1.65E-13 | 1 | -0.40 | 2.43 | Nurse High |  |
| *GB19783* |  | 6.13E-05 | 3.02E-02 | 0.50 | -1.62 | 1.30 | Nurse High |  |
| *GB19799* | *vanin-like* | 1.00E+00 | 6.78E-02 | 1 | 0.13 | 0.70 |  |  |
| *GB19866* |  | 1.00E+00 | 3.71E-03 | 1 | -0.03 | 0.59 |  |  |
| *GB19924* |  | 1.00E+00 | 1.45E-02 | 1 | -0.59 | -1.60 |  |  |
| *GB19937* | *SoxN* | 1.77E-01 | 2.12E-04 | 1 | 1.58 | 2.19 |  | Yes |
| *GB20061* |  | 8.21E-02 | 3.38E-02 | 1 | 0.79 | 1.03 |  |  |
| *GB30149* |  | 3.66E-01 | 1.51E-04 | 1 | 2.63 | Inf |  |  |
| *GB30312* |  | 1.00E+00 | 2.31E-08 | 0.52 | -0.61 | 1.85 | Nurse High |  |
| *GB30366* |  | 6.67E-01 | 6.07E-02 | 1 | -0.42 | 0.70 | Forager High |  |
| *GB30578* |  | 4.18E-06 | 1.01E-05 | 1 | 2.51 | 2.28 |  |  |

Table S2. Genes differentially expressed in response to juvenile hormone analog treatment.

| ***ID*** | ***D.mel. Ortholog*** | ***p.adj(JHA)*** | ***p.adj(USP)*** | ***p.adj(USPxJHA)*** | ***log2(JHA-Control)*** | ***log2(dsGFP-dsUSP)*** | ***Maturation*** | ***ChIP*** |
| --- | --- | --- | --- | --- | --- | --- | --- | --- |
| *GB10157* | *CG31743* | 7.58E-02 | 1.00E+00 | 1 | 1.13 | 0.61 |  |  |
| *GB10209* | *CG8927* | 1.03E-03 | 1.00E+00 | 1 | -0.71 | -0.32 | Nurse High |  |
| *GB10222* | *CG31954* | 2.73E-08 | 2.65E-01 | 1 | 4.28 | 1.70 | Forager High | |
| *GB10252* | *CG6597* | 9.42E-02 | 4.48E-02 | 1 | 0.74 | 0.74 |  |  |
| *GB10330* |  | 3.26E-10 | 1.00E+00 | 1 | 1.43 | 0.42 |  |  |
| *GB10355* |  | 3.28E-03 | 1.00E+00 | 1 | -1.13 | 0.81 | Nurse High |  |
| *GB10428* | *CG33998* | 1.73E-12 | 7.89E-06 | 1 | 2.01 | 1.70 | Forager High | |
| *GB10517* | *jhamt* | 1.05E-02 | 1.00E+00 | 1 | 1.55 | 0.25 |  | Yes |
| *GB10536* |  | 1.20E-02 | 6.79E-01 | 1 | -0.84 | -0.27 |  |  |
| *GB10655* |  | 1.50E-02 | 2.93E-01 | 1 | 0.60 | -0.56 |  |  |
| *GB10676* |  | 0.00E+00 | 1.00E+00 | 1 | -1.51 | -0.28 | Nurse High |  |
| *GB10764* |  | 4.22E-02 | 9.05E-03 | 1 | -1.55 | 2.08 |  |  |
| *GB10774* | *inx7* | 2.35E-05 | 1.00E+00 | 1 | 1.17 | 0.64 | Forager High | |
| *GB10794* | *unc-5* | 8.52E-02 | 2.27E-01 | 1 | -0.49 | -0.46 |  |  |
| *GB10795* | *CG8839* | 1.08E-02 | 1.00E+00 | 1 | -0.62 | -0.02 | Nurse High |  |
| *GB10820* |  | 1.42E-09 | 1.25E-01 | 1 | -2.15 | 1.12 |  |  |
| *GB10971* |  | 5.37E-09 | 5.22E-01 | 1 | 1.15 | 0.41 |  |  |
| *GB11027* |  | 2.95E-02 | 1.00E+00 | 1 | -0.61 | -0.05 |  |  |
| *GB11092* | *Obp56g* | 5.68E-09 | 1.00E+00 | 1 | -1.09 | -0.25 | Nurse High |  |
| *GB11111* |  | 8.28E-03 | 2.04E-02 | 1 | 1.81 | 1.48 |  |  |
| *GB11149* |  | 4.78E-02 | 1.00E+00 | 1 | 0.91 | 0.66 |  | Yes |
| *GB11204* | *daw* | 4.56E-04 | 1.00E+00 | 1 | -0.74 | 0.03 |  |  |
| *GB11240* | *CG5508* | 5.01E-02 | 1.00E+00 | 1 | -0.53 | 0.05 | Nurse High |  |
| *GB11256* |  | 1.04E-07 | 7.93E-05 | 1 | -1.29 | 1.04 |  |  |
| *GB11259* | *CG31689* | 1.85E-12 | 1.00E+00 | 1 | -1.43 | -0.17 | Nurse High |  |
| *GB11360* |  | 9.56E-03 | 1.00E+00 | 1 | 0.53 | 0.26 |  | Yes |
| *GB11384* | *CG13566* | 9.81E-03 | 1.00E+00 | 1 | -0.66 | 0.20 |  |  |
| *GB11412* | *CG1443* | 8.53E-11 | 5.05E-01 | 1 | -1.24 | -0.45 | Nurse High |  |
| *GB11581* | *Adgf-A* | 2.46E-03 | 2.19E-03 | 1 | 1.02 | 0.92 |  |  |
| *GB11808* | *CG6928* | 6.96E-02 | 1.00E+00 | 1 | -0.60 | -0.21 |  |  |
| *GB11812* |  | 9.14E-07 | 1.00E+00 | 0.31 | 0.90 | -0.24 |  |  |
| *GB11834* | *CG6356* | 8.52E-02 | 4.25E-01 | 1 | 0.89 | 0.72 |  |  |
| *GB11866* |  | 4.90E-02 | 1.00E+00 | 1 | -1.59 | -0.25 |  |  |
| *GB11908* |  | 1.81E-05 | 1.00E+00 | 1 | -1.23 | -0.18 | Nurse High |  |
| *GB11929* |  | 5.17E-05 | 1.00E+00 | 1 | -0.81 | -0.28 | Nurse High |  |
| *GB11943* | *Cyp305a1* | 0.00E+00 | 4.61E-01 | 1 | 1.63 | 0.19 |  |  |
| *GB12054* |  | 6.19E-04 | 1.00E+00 | 1 | 0.76 | 0.17 | Forager High | |
| *GB12096* |  | 2.03E-02 | 1.00E+00 | 1 | -1.03 | -0.54 |  |  |
| *GB12121* |  | 0.00E+00 | 1.00E+00 | 1 | -1.78 | -0.19 | Nurse High |  |
| *GB12135* | *CG1213* | 1.34E-04 | 6.67E-01 | 1 | 1.72 | 0.96 |  |  |
| *GB12136* |  | 1.60E-02 | 1.45E-03 | 1 | -4.22 | Inf |  |  |
| *GB12136* |  | 1.60E-02 | 1.45E-03 | 1 | -4.22 | Inf |  |  |
| *GB12158* |  | 2.36E-02 | 1.62E-04 | 1 | 0.11 | 1.26 | Nurse High |  |
| *GB12176* | *CG5326* | 0.00E+00 | 1.00E+00 | 1 | -1.79 | -0.16 | Nurse High |  |
| *GB12190* |  | 3.64E-05 | 1.00E+00 | 1 | -1.39 | 0.41 | Nurse High |  |
| *GB12203* |  | 6.58E-11 | 2.17E-04 | 1 | 2.69 | 1.32 | Forager High | |
| *GB12259* | *CG42249* | 9.55E-02 | 1.18E-03 | 1 | 0.74 | 1.16 |  |  |
| *GB12292* |  | 4.78E-02 | 7.98E-02 | 1 | -1.58 | 1.77 | Nurse High |  |
| *GB12319* |  | 4.58E-02 | 1.00E+00 | 1 | -0.58 | 0.16 |  |  |
| *GB12340* |  | 0.00E+00 | 1.75E-03 | 1 | -1.38 | -0.62 | Nurse High |  |
| *GB12353* |  | 2.77E-06 | 1.91E-02 | 1 | 2.45 | 1.42 | Forager High | |
| *GB12607* |  | 1.29E-06 | 5.50E-03 | 1 | 2.81 | 1.22 | Forager High | |
| *GB12679* | *CG9812* | 6.13E-05 | 1.00E+00 | 1 | -0.86 | -0.24 | Nurse High |  |
| *GB12745* | *CG13196* | 4.36E-08 | 4.31E-02 | 1 | -1.87 | -1.02 |  |  |
| *GB12751* |  | 3.68E-12 | 1.00E+00 | 1 | -1.56 | -0.23 | Nurse High |  |
| *GB12896* |  | 3.62E-03 | 1.71E-05 | 1 | 0.97 | 1.36 | Forager High | |
| *GB13049* |  | 5.35E-03 | 1.00E+00 | 1 | -0.83 | 0.18 |  |  |
| *GB13052* |  | 6.17E-03 | 1.00E+00 | 1 | -0.72 | 0.02 | Nurse High |  |
| *GB13208* |  | 3.19E-02 | 1.00E+00 | 1 | -0.58 | -0.06 |  |  |
| *GB13238* |  | 2.72E-03 | 4.81E-01 | 1 | 0.73 | 0.93 |  |  |
| *GB13285* |  | 2.13E-05 | 1.80E-01 | 1 | -1.40 | 0.99 | Nurse High |  |
| *GB13351* | *sPLA2* | 8.21E-02 | 2.45E-01 | 1 | -1.22 | 0.95 | Nurse High |  |
| *GB13365* |  | 1.32E-04 | 5.63E-01 | 1 | -2.11 | -1.18 | Nurse High |  |
| *GB13473* |  | 1.63E-05 | 1.00E+00 | 1 | -1.26 | 0.68 |  |  |
| *GB13473* |  | 5.15E-04 | 1.00E+00 | 1 | -1.20 | 0.85 |  |  |
| *GB13473* |  | 9.52E-06 | 1.00E+00 | 1 | -1.18 | 0.70 |  |  |
| *GB13473* |  | 1.63E-05 | 1.00E+00 | 1 | -1.26 | 0.68 |  |  |
| *GB13473* |  | 1.63E-05 | 1.00E+00 | 1 | -1.26 | 0.68 |  |  |
| *GB13473* |  | 1.63E-05 | 1.00E+00 | 1 | -1.26 | 0.68 | Forager High | |
| *GB13473* |  | 1.63E-05 | 1.00E+00 | 1 | -1.26 | 0.68 |  |  |
| *GB13473* |  | 5.15E-04 | 1.00E+00 | 1 | -1.20 | 0.85 |  |  |
| *GB13473* |  | 5.15E-04 | 1.00E+00 | 1 | -1.20 | 0.85 | Forager High | |
| *GB13473* |  | 5.15E-04 | 1.00E+00 | 1 | -1.20 | 0.85 |  |  |
| *GB13473* |  | 5.15E-04 | 1.00E+00 | 1 | -1.20 | 0.85 |  |  |
| *GB13473* |  | 9.52E-06 | 1.00E+00 | 1 | -1.18 | 0.70 |  |  |
| *GB13473* |  | 9.52E-06 | 1.00E+00 | 1 | -1.18 | 0.70 | Forager High | |
| *GB13473* |  | 9.52E-06 | 1.00E+00 | 1 | -1.18 | 0.70 |  |  |
| *GB13473* |  | 9.52E-06 | 1.00E+00 | 1 | -1.18 | 0.70 |  |  |
| *GB13507* | *CG10623* | 8.94E-03 | 1.00E+00 | 1 | -0.62 | 0.01 | Nurse High |  |
| *GB13546* |  | 4.78E-02 | 1.49E-04 | 1 | 2.53 | 1.29 |  |  |
| *GB13591* |  | 3.91E-05 | 1.00E+00 | 1 | 1.38 | -0.02 |  |  |
| *GB13619* | *CG15343* | 2.06E-03 | 1.00E+00 | 1 | 0.73 | -0.32 | Forager High | |
| *GB13620* | *CG15533* | 1.43E-02 | 2.61E-03 | 1 | 1.34 | 1.62 |  |  |
| *GB13621* | *CG32250* | 4.47E-05 | 1.00E+00 | 1 | -0.84 | -0.20 | Nurse High |  |
| *GB13633* |  | 1.91E-02 | 4.76E-01 | 1 | -1.04 | 0.78 |  |  |
| *GB13695* | *CG1441* | 1.75E-02 | 1.00E+00 | 1 | -0.75 | -0.13 |  |  |
| *GB13764* | *CG15658* | 2.19E-02 | 1.00E+00 | 1 | 0.72 | 0.47 |  |  |
| *GB13833* | *Tsp* | 9.82E-03 | 1.00E+00 | 1 | 0.59 | -0.11 | Forager High | |
| *GB13859* |  | 4.44E-03 | 1.00E+00 | 1 | 0.65 | -0.12 | Forager High | |
| *GB13921* | *CG33970* | 1.39E-08 | 1.00E+00 | 1 | -1.07 | -0.04 | Nurse High |  |
| *GB13930* | *CG1946* | 5.19E-10 | 1.00E+00 | 1 | -1.16 | 0.01 | Nurse High |  |
| *GB13999* |  | 2.27E-03 | 1.00E+00 | 1 | -1.48 | 0.09 | Nurse High |  |
| *GB14001* |  | 1.12E-04 | 1.00E+00 | 1 | 1.13 | 0.70 | Forager High | |
| *GB14026* | *inx2* | 2.64E-02 | 8.30E-01 | 1 | 0.71 | 0.42 |  |  |
| *GB14105* | *Oatp58Dc* | 1.74E-02 | 6.32E-01 | 1 | 0.39 | 0.61 |  |  |
| *GB14149* | *Cpr97Ea* | 6.46E-03 | 1.00E+00 | 1 | -0.63 | 0.25 |  | Yes |
| *GB14169* |  | 4.60E-07 | 8.50E-04 | 1 | 1.52 | 1.28 |  |  |
| *GB14261* | *Npc2d* | 1.67E-02 | 1.08E-11 | 0.43 | 0.66 | 1.68 | Nurse High |  |
| *GB14361* |  | 2.48E-03 | 8.00E-01 | 1 | -0.59 | 0.48 | Nurse High |  |
| *GB14396* |  | 1.79E-02 | 1.00E+00 | 1 | -0.66 | -0.02 | Nurse High |  |
| *GB14528* | *CG2680* | 2.17E-03 | 1.00E+00 | 1 | 0.84 | -0.22 |  |  |
| *GB14603* | *CG16996* | 3.08E-04 | 1.12E-07 | 0.74 | 1.52 | 1.53 | Nurse High |  |
| *GB14612* |  | 3.66E-04 | 1.00E+00 | 1 | 0.75 | 0.08 | Forager High | |
| *GB14638* | *dyl* | 2.42E-02 | 1.00E+00 | 1 | -0.89 | 0.00 |  | Yes |
| *GB14667* |  | 8.05E-02 | 1.03E-01 | 1 | 1.26 | 1.36 |  |  |
| *GB14761* |  | 5.03E-02 | 1.00E+00 | 1 | 0.59 | 0.38 | Forager High | |
| *GB14784* | *CG15712* | 4.52E-06 | 9.03E-08 | 0.87 | 1.20 | 1.27 | Nurse High | Yes |
| *GB14861* | *CG6938* | 3.00E-02 | 1.00E+00 | 1 | -0.80 | 0.31 |  |  |
| *GB14913* |  | 8.77E-04 | 2.56E-01 | 1 | -0.98 | 0.82 | Nurse High |  |
| *GB14975* | *CG3244* | 6.05E-03 | 1.12E-01 | 1 | -0.63 | -0.51 | Nurse High |  |
| *GB15246* | *CG13842* | 6.15E-02 | 9.82E-02 | 1 | 2.03 | 1.30 |  |  |
| *GB15303* | *ple* | 8.43E-02 | 3.45E-01 | 1 | 0.50 | 0.45 |  |  |
| *GB15323* | *CG34115* | 3.48E-02 | 1.00E+00 | 1 | -0.59 | 0.06 | Nurse High |  |
| *GB15452* | *CG13510* | 7.66E-03 | 1.00E+00 | 1 | 0.70 | 0.24 | Forager High | |
| *GB15460* |  | 9.48E-03 | 1.00E+00 | 1 | -0.71 | -0.01 | Nurse High |  |
| *GB15518* | *CG9380* | 2.04E-06 | 3.91E-04 | 1 | 1.14 | 0.98 |  |  |
| *GB15672* |  | 3.38E-05 | 1.00E+00 | 0.15 | 0.58 | 0.67 | Forager High | |
| *GB15934* |  | 6.18E-02 | 1.00E+00 | 1 | 0.91 | 0.36 | Forager High | |
| *GB16165* | *mwh* | 6.25E-05 | 4.64E-01 | 1 | -0.88 | -0.47 |  |  |
| *GB16262* |  | 3.10E-05 | 1.00E+00 | 1 | 2.41 | 0.64 |  |  |
| *GB16388* |  | 9.19E-07 | 1.00E+00 | 1 | -1.09 | -0.04 | Nurse High |  |
| *GB16399* |  | 4.13E-07 | 1.25E-05 | 1 | 1.59 | 1.57 | Forager High | |
| *GB16536* |  | 2.71E-02 | 4.82E-02 | 1 | 0.72 | -0.67 |  |  |
| *GB16672* | *CG9170* | 4.27E-04 | 1.00E+00 | 1 | 0.85 | 0.50 |  |  |
| *GB16692* |  | 5.08E-03 | 1.00E+00 | 1 | -0.65 | 0.05 | Nurse High |  |
| *GB16692* | *Cpr100A* | 5.08E-03 | 1.00E+00 | 1 | -0.65 | 0.05 | Nurse High |  |
| *GB16709* |  | 4.78E-02 | 1.08E-01 | 1 | 1.31 | 1.06 |  |  |
| *GB16730* | *CG42351* | 3.66E-04 | 1.00E+00 | 1 | -0.90 | -0.30 | Nurse High |  |
| *GB16731* | *CG11409* | 1.20E-02 | 1.00E+00 | 1 | -0.89 | 0.24 |  |  |
| *GB16732* |  | 1.05E-05 | 2.31E-05 | 1 | 2.04 | 1.59 |  | Yes |
| *GB16747* | *CG17323* | 4.95E-05 | 1.00E+00 | 1 | 0.85 | 0.10 | Forager High | |
| *GB16769* |  | 6.25E-05 | 7.30E-04 | 1 | 2.19 | 1.87 |  |  |
| *GB16807* |  | 2.19E-02 | 1.00E+00 | 1 | -0.58 | -0.16 | Nurse High |  |
| *GB16848* |  | 1.99E-07 | 1.00E+00 | 1 | 0.91 | 0.15 | Forager High | |
| *GB16884* |  | 3.17E-02 | 1.00E+00 | 1 | -0.63 | 0.16 | Nurse High |  |
| *GB16948* |  | 2.06E-03 | 1.00E+00 | 1 | -0.69 | -0.09 |  |  |
| *GB17011* |  | 1.19E-02 | 4.82E-02 | 1 | 1.20 | 1.20 |  |  |
| *GB17015* | *Ugt86Dg* | 3.66E-04 | 1.00E+00 | 1 | 1.04 | -0.11 | Forager High | |
| *GB17017* | *Oatp33Eb* | 3.49E-08 | 8.49E-08 | 0.51 | 1.56 | 1.49 |  |  |
| *GB17166* | *MESR3* | 1.17E-07 | 7.60E-04 | 1 | 2.19 | 1.66 |  |  |
| *GB17310* |  | 9.51E-02 | 1.00E+00 | 1 | 0.51 | 0.05 | Forager High | |
| *GB17341* | *snky* | 3.75E-04 | 1.00E+00 | 1 | -1.16 | 0.55 |  |  |
| *GB17345* | *CG4998* | 4.76E-02 | 1.61E-01 | 1 | -0.45 | 0.56 |  |  |
| *GB17354* |  | 6.25E-08 | 1.00E+00 | 1 | -1.23 | 0.75 |  |  |
| *GB17418* | *Calx* | 5.18E-02 | 6.67E-01 | 1 | 2.18 | -1.66 |  |  |
| *GB17434* |  | 2.71E-02 | 1.00E+00 | 1 | 0.68 | 0.14 | Forager High | |
| *GB17481* | *CG11626* | 1.78E-05 | 2.65E-07 | 0.09 | 1.18 | 1.34 |  |  |
| *GB17538* |  | 1.01E-02 | 1.00E+00 | 1 | -0.97 | 0.59 | Forager High | |
| *GB17588* |  | 1.61E-03 | 4.95E-01 | 1 | -0.77 | -0.55 | Forager High | |
| *GB17638* |  | 5.67E-04 | 1.00E+00 | 1 | -1.18 | 0.06 | Forager High | |
| *GB17663* |  | 2.42E-02 | 1.00E+00 | 1 | -0.73 | 0.02 | Nurse High |  |
| *GB17782* |  | 4.93E-04 | 1.00E+00 | 1 | -1.12 | 0.63 |  |  |
| *GB17782* |  | 4.93E-04 | 1.00E+00 | 1 | -1.12 | 0.63 |  |  |
| *GB17782* |  | 4.93E-04 | 1.00E+00 | 1 | -1.12 | 0.63 |  |  |
| *GB17782* |  | 4.93E-04 | 1.00E+00 | 1 | -1.12 | 0.63 | Forager High | |
| *GB17782* |  | 4.93E-04 | 1.00E+00 | 1 | -1.12 | 0.63 |  |  |
| *GB17798* |  | 1.15E-04 | 1.00E+00 | 1 | 0.86 | 0.26 |  |  |
| *GB18032* |  | 2.42E-02 | 1.00E+00 | 1 | -1.04 | -0.08 |  |  |
| *GB18059* | *CG6746* | 6.75E-03 | 1.00E+00 | 1 | -0.56 | -0.25 | Nurse High |  |
| *GB18123* | *mp* | 7.07E-02 | 1.00E+00 | 1 | 0.59 | 0.17 |  |  |
| *GB18161* |  | 1.46E-08 | 2.12E-04 | 1 | -1.83 | 1.42 | Nurse High |  |
| *GB18264* |  | 2.27E-02 | 1.45E-04 | 1 | 1.25 | 1.83 | Forager High | |
| *GB18330* |  | 9.63E-03 | 1.00E+00 | 1 | 0.67 | 0.07 |  |  |
| *GB18334* |  | 0.00E+00 | 3.91E-08 | 0.50 | 2.38 | 1.10 | Forager High | |
| *GB18360* | *CG11796* | 2.32E-02 | 9.21E-03 | 1 | 0.87 | 0.85 | Forager High | |
| *GB18472* |  | 1.35E-03 | 1.00E+00 | 1 | -1.03 | -0.36 | Nurse High |  |
| *GB18507* | *stai* | 2.91E-04 | 1.00E+00 | 1 | 1.00 | 0.06 |  |  |
| *GB18543* |  | 0.00E+00 | 1.00E+00 | 1 | -1.78 | 0.65 | Nurse High |  |
| *GB18552* |  | 1.00E+00 | 1.00E+00 | 1 | 0.12 | -0.13 |  | Yes |
| *GB18552* |  | 3.15E-03 | 1.00E+00 | 1 | -1.13 | -0.24 |  | Yes |
| *GB18562* | *CG10026* | 1.27E-03 | 1.00E+00 | 1 | -0.76 | -0.05 | Nurse High |  |
| *GB18562* |  | 1.27E-03 | 1.00E+00 | 1 | -0.76 | -0.05 | Nurse High |  |
| *GB18599* | *CG6287* | 2.48E-03 | 1.00E+00 | 1 | -1.16 | -0.34 |  |  |
| *GB18743* |  | 6.05E-03 | 1.00E+00 | 1 | 0.61 | -0.16 |  |  |
| *GB18762* |  | 1.73E-12 | 6.16E-01 | 1 | -1.48 | -0.47 | Nurse High |  |
| *GB18767* | *CG9372* | 3.19E-02 | 1.00E+00 | 1 | -0.68 | 0.01 | Nurse High |  |
| *GB18803* |  | 2.94E-03 | 1.00E+00 | 1 | 0.67 | 0.21 |  |  |
| *GB18896* | *CG9701* | 1.54E-02 | 1.00E+00 | 1 | 0.81 | -0.23 | Forager High | |
| *GB18947* |  | 9.45E-03 | 1.45E-03 | 1 | 0.86 | 0.96 |  | Yes |
| *GB19006* | *CG3884* | 7.72E-04 | 9.61E-02 | 1 | 1.13 | 0.95 | Forager High | |
| *GB19027* | *CG3277* | 6.29E-02 | 3.31E-01 | 1 | 1.67 | 1.62 | Nurse High |  |
| *GB19044* |  | 4.15E-02 | 1.00E+00 | 1 | 4.11 | -0.29 |  |  |
| *GB19099* |  | 4.23E-08 | 4.87E-05 | 1 | 1.74 | 1.42 |  |  |
| *GB19107* | *CG7365* | 0.00E+00 | 1.00E+00 | 1 | -1.55 | -0.33 | Nurse High |  |
| *GB19135* |  | 1.40E-02 | 1.00E+00 | 1 | 0.64 | -0.23 |  |  |
| *GB19262* |  | 4.09E-02 | 1.00E+00 | 1 | 5.16 | 0.62 |  |  |
| *GB19264* | *per* | 8.40E-03 | 1.00E+00 | 1 | 0.62 | 0.13 |  |  |
| *GB19265* |  | 8.28E-03 | 1.00E+00 | 1 | -0.79 | -0.13 |  | Yes |
| *GB19268* |  | 4.97E-09 | 1.00E+00 | 1 | -1.29 | 0.03 | Nurse High |  |
| *GB19322* |  | 5.35E-02 | 1.00E+00 | 1 | 0.45 | 0.18 |  |  |
| *GB19401* |  | 6.48E-05 | 3.58E-04 | 1 | 2.49 | 2.20 |  |  |
| *GB19418* |  | 1.22E-06 | 1.00E+00 | 1 | 1.26 | -0.34 |  |  |
| *GB19418* |  | 1.22E-06 | 1.00E+00 | 1 | 1.26 | -0.34 |  |  |
| *GB19418* |  | 4.36E-08 | 3.98E-01 | 1 | 1.58 | -0.60 |  |  |
| *GB19418* |  | 4.36E-08 | 3.98E-01 | 1 | 1.58 | -0.60 |  |  |
| *GB19617* |  | 1.57E-02 | 4.22E-01 | 1 | 1.38 | 0.89 |  |  |
| *GB19643* |  | 9.92E-04 | 4.76E-02 | 1 | 0.94 | 0.98 |  |  |
| *GB19683* | *emp* | 1.02E-06 | 1.00E+00 | 1 | -0.95 | -0.26 | Nurse High |  |
| *GB19683* | *emp* | 1.09E-03 | 1.00E+00 | 1 | -0.72 | -0.20 | Nurse High |  |
| *GB19735* | *Npc2a* | 2.71E-02 | 1.00E+00 | 1 | 0.56 | 0.11 | Forager High | |
| *GB19779* | *CG42513* | 9.76E-02 | 1.00E+00 | 1 | 0.49 | -0.03 | Forager High | |
| *GB19783* |  | 6.13E-05 | 3.02E-02 | 0.50 | -1.62 | 1.30 | Nurse High |  |
| *GB19894* |  | 1.37E-02 | 1.00E+00 | 1 | 0.91 | -0.26 |  |  |
| *GB19935* |  | 1.77E-02 | 1.00E+00 | 1 | -0.44 | 0.20 | Nurse High |  |
| *GB20048* | *CG33281* | 7.49E-02 | 1.00E+00 | 1 | 0.44 | 0.54 | Forager High | Yes |
| *GB20051* |  | 8.83E-03 | 1.00E+00 | 1 | 3.44 | -0.55 |  | Yes |
| *GB20061* |  | 8.21E-02 | 3.38E-02 | 1 | 0.79 | 1.03 |  |  |
| *GB30070* |  | 7.49E-02 | 1.00E+00 | 1 | -0.63 | -0.26 |  | Yes |
| *GB30070* |  | 7.49E-02 | 1.00E+00 | 1 | -0.63 | -0.26 |  | Yes |
| *GB30070* |  | 7.49E-02 | 1.00E+00 | 1 | -0.63 | -0.26 |  | Yes |
| *GB30070* |  | 7.49E-02 | 1.00E+00 | 1 | -0.63 | -0.26 |  | Yes |
| *GB30090* |  | 9.81E-03 | 1.00E+00 | 1 | 0.63 | -0.26 |  |  |
| *GB30090* |  | 2.87E-02 | 1.00E+00 | 1 | 0.54 | -0.10 |  |  |
| *GB30090* |  | 9.81E-03 | 1.00E+00 | 1 | 0.63 | -0.26 | Forager High | |
| *GB30090* |  | 2.87E-02 | 1.00E+00 | 1 | 0.54 | -0.10 | Forager High | |
| *GB30090* |  | 8.28E-03 | 9.94E-01 | 1 | 0.60 | -0.35 |  |  |
| *GB30090* |  | 8.28E-03 | 9.94E-01 | 1 | 0.60 | -0.35 | Forager High | |
| *GB30200* | *Cpr97Eb* | 3.17E-03 | 1.00E+00 | 1 | -0.86 | 0.16 |  |  |
| *GB30204* |  | 4.53E-03 | 1.00E+00 | 1 | -0.86 | 0.20 | Nurse High | Yes |
| *GB30243* |  | 2.74E-03 | 1.00E+00 | 1 | -3.60 | -0.78 |  |  |
| *GB30317* |  | 7.53E-04 | 1.00E+00 | 1 | 0.68 | -0.19 | Nurse High |  |
| *GB30317* |  | 7.53E-04 | 1.00E+00 | 1 | 0.68 | -0.19 | Nurse High |  |
| *GB30365* |  | 5.57E-03 | 4.64E-01 | 0.33 | -0.84 | -0.45 | Nurse High |  |
| *GB30439* |  | 5.01E-02 | 1.00E+00 | 1 | Inf | 1.44 |  |  |
| *GB30473* |  | 2.13E-05 | 1.00E+00 | 1 | 1.42 | 0.31 |  |  |
| *GB30474* |  | 2.73E-08 | 5.05E-01 | 1 | 1.43 | 0.37 | Forager High | |
| *GB30494* |  | 8.94E-03 | 1.00E+00 | 1 | -0.61 | 0.19 | Forager High | |
| *GB30556* | *Prestin* | 4.01E-04 | 1.00E+00 | 1 | 1.44 | -0.08 |  |  |
| *GB30578* |  | 4.18E-06 | 1.01E-05 | 1 | 2.51 | 2.28 |  |  |
